# Proteasome inhibition as a potential therapeutic target in thymic cancer

**DOI:** 10.1038/s41419-025-08240-5

**Published:** 2025-12-04

**Authors:** Satoru Okada, Louisa Benter, Leon Schrell, Denise Müller, Selen Selcen, Hanibal Bohnenberger, Carolin Schneider, Günter Schneider, Melanie Lohrberg, Raphael Koch, Tobias R. Overbeck, Alexander von Hammerstein-Equord, Stefan Welter, Marc Hinterthaner, Lucia Cordes, Katayoon Shirneshan, Christoph Netzer, Masayoshi Inoue, Alexander Marx, Philipp Ströbel, Stefan Küffer

**Affiliations:** 1 Institute of Pathology, University Medical Center Göttingen, University of Göttingen, Göttingen, Germany; 2 Division of Thoracic Surgery, Department of Surgery, Graduate School of Medical Science, Kyoto Prefectural University of Medicine, Kyoto, Japan; 3 Department of General, Visceral and Pediatric Surgery, University Medical Center Göttingen, Göttingen, Germany; 4 CCC-N (Comprehensive Cancer Center Lower Saxony), Göttingen, Germany; 5 Department of Haematology and Medical Oncology, University Medical Centre Göttingen, Göttingen, Germany; 6 Department of Thoracic and Cardiovascular Surgery, University Medical Center, Göttingen, Germany; 7 Thoracic Surgery Department, Lung Clinic Hemer, Hemer, Germany; 8 Department of Otorhinolaryngology, Head and Neck Surgery, University Medical Center Göttingen, Göttingen, Germany; 9 Department of Otorhinolaryngology, Head and Neck Surgery, Heidelberg University Hospital, Heidelberg, Germany

**Keywords:** Preclinical research, Cancer metabolism

## Abstract

Multimodal radio-chemotherapy is the mainstay of treatment for unresectable thymoma (TH) and thymic carcinoma (TC), but there is an urgent need for other therapeutic strategies in these rare tumors. The epithelial cells of the normal thymus express the three major proteasome classes: constitutive, immunoproteasome, and thymoproteasome, making thymic epithelial tumors potential candidates for treatment with proteasome inhibitors. In a drug screen of 120 cytotoxic agents, the two thymic carcinoma cell lines 1889c and MP57 showed exquisite sensitivity to the proteasome inhibitor carfilzomib (PR-171). Immunohistochemistry, gene expression, and in vitro functional studies were used in a comprehensive sample collection to investigate the correlation between immunoproteasome subunit expression and response to carfilzomib. 50% of TC and a substantial proportion of TH strongly expressed immunoproteasome subunits and showed functional activity of β1i (PSMB9), β2i (PSMB10), and β5i (PSMB8). INF-γ treatment induced immunoproteasome expression and increased cell sensitivity to carfilzomib, while siRNA knockdown reduced carfilzomib response in vitro. Carfilzomib synergized with BCL2 family protein inhibitors (navitoclax or AZD5991), suggesting that drug combinations could be used to reduce the dose of each drug to minimize toxicity. Notably, thymic carcinomas differed from squamous cell carcinomas in other organs by higher levels of β5i (PSMB8) and constitutive proteasome β5 (PSMB5). We hypothesize that TC (and probably many TH) are uniquely suited for treatment with proteasome inhibitors alone or in combination with selective BH3 mimetics.

## Introduction

Thymomas (TH) and thymic carcinomas (TC) are rare thymic epithelial tumors (TET). The World Health Organization (WHO) employs a classification for TH encompassing five primary categories: A, AB, B1, B2, and B3. This system is based on the morphology of the neoplastic epithelial cells and the proportion of immature lymphocytes [1]. Radical surgery represents the sole curative treatment for TET; however, advanced TET frequently necessitate a multimodal approach incorporating radio-chemotherapy. Comprehensive genomic analyses have revealed one of the lowest tumor mutational burdens (TMB) of all adult cancers and have failed to detect oncogenic driver mutations in the majority of cases [2]. Consequently, except for imatinib in the rare TC cases with *KIT* mutations [3] and immunotherapy for PD-L1 positive TC [4, 5], no other TET therapies are based on defined molecular targets or experimentally validated data.

Proteasome inhibition has been successfully employed as a therapeutic strategy for multiple myeloma. It has also been utilized in the treatment of several solid tumors, including hepatocellular carcinoma, non-small cell lung cancer, glioblastoma, and neuroblastoma [6–8]. The ubiquitin-proteasome system (UPS) is expressed in all cells and serves to regulate proteostasis by facilitating the degradation of unwanted, misfolded, and foreign proteins. In tumor cells, the UPS can facilitate tumor growth by interfering with cell signaling pathways, immune regulation, and drug resistance [8], simultaneously creating a potential vulnerability. Inhibiting the UPS in cancer cells results in excessive protein overload and induces apoptosis and inflammation [9].

The UPS consists of three distinct subtypes: the constitutive proteasome (CPS), the immunoproteasome (IPS), and the thymoproteasome (TPS). CPS is primarily responsible for protein turnover, while the IPS and the TPS have a specific role in antigen presentation through the major histocompatibility complex class I on professional antigen-presenting cells [10] and thymic epithelial cells [11]. Expression of the TPS is restricted to cortical thymic epithelial cells (cTEC) but is also found in many thymomas[12, 13]. The CPS expresses the β1, β2, and β5 subunits (PSMB6, PSMB7, and PSMB5), whereas the immunoproteasome expresses the subunits β1i (PSMB9), β2i (PSMB10) and β5i (PSMB8) subunits that are homologous to those expressed by the CPS. The IPS and the TPS share β1i (PSMB9) and β2i (PSMB10) and differ only by the expression of β5t (PSMB11) (PMID: 35159231). However, the expression and function of the CPS and IPS in TET have not been studied in detail.

Screening of 120 FDA-approved drugs in two thymic carcinoma cell lines (1889c and MP57) identified the proteasome inhibitor carfilzomib and the ubiquitinase inhibitor TAK-243 as highly efficacious drugs at low nanomolar concentrations. A comprehensive analysis of the proteasome was conducted in 138 TET samples through immunohistochemical (IHC) staining and gene expression profiling using 115 TH and TC samples from the Cancer Genome Atlas (TCGA).

The present study demonstrates that carfilzomib inhibits the CPS and the IPS, induces apoptosis, and acts synergistically with the antiapoptotic drugs navitoclax and AZD5991. Based on these mechanisms, we propose that proteasome inhibition (e.g., by carfilzomib) alone or combined with other drugs represents a potential new therapeutic approach for treating non-resectable or recurrent TC.

## Materials and methods

### Clinical patient data and tissues

All TET samples were classified according to the most recent WHO classification, and the modified Masaoka–Koga classification assessed the tumor stage. The native tissue specimens of TET, lung squamous cell carcinomas (LSCC), and head and neck squamous cell carcinomas (HNSCC) were provided by the Department of Thoracic and Cardiovascular Surgery, the Department of Otorhinolaryngology, and the Department of Head and Neck Surgery of the University Medical Center Göttingen. The tissues were rapidly frozen in liquid nitrogen and stored at -80°C until further processing. All procedures were conducted in accordance with the seventh edition of the Declaration of Helsinki. The project was approved by the ethics committee of the University Medical Center Göttingen (reference number GÖ 912/15) (Table 1).

**Table 1:** Clinical data summary.

		TH/TC (*n* = 138)
Sex	Male	54 (39.1%)
	Female	76 (55.1%)
	Unknown	8 (5.8%)
Age (y)	Median (IQR)	62 (51–69)
	Range	28-88
Type	A	12 (8.7%)
	AB	45 (32.6%)
	B1	17 (12.3%)
	B2	35 (25.4%)
	B3	19 (13.8%)
	C	10 (7.2%)
Masaoka-Koga	1	30 (21.7%)
	2	50 (36.2%)
	3	23 (16.7%)
	4	10 (7.2%)
	Unknown	25 (18.1%)
Follow up (month)	Median (IQR)	60 (30–93)
	Range	1-252
Death		14

*IQR* interquartile range, *WHO* World Health Organization.

Survival information was available for 47 patients, including 28 patients with B2 and B3 thymomas and thymic carcinomas.

### Drug Library and screening of 1889c and MP57

The compound library was cherry-picked from Selleck Chemicals. The screening was conducted as previously described [14]. In brief, 2000 cells per well were seeded in a 96-well plate (3610, Corning Life Sciences). Drug screening was performed as a single biological replicate performed as technical duplicates. After 24 hours of incubation, cells were treated with the diluted compound library for 72 hours. The TC cell lines 1889c (courtesy of Ehemann et al. [15]) and MP57 (courtesy of Giaccone et al. [16]) were treated with 7 concentrations of each compound to attain the following final treatment concentrations: 10, 3.3, 1.1, 0.37, 0.12, 0.04, and 0.014 µm and DMSO as control. ATP was quantified as a surrogate for the dose response using the CellTiter-Glo assay (Promega). The area under the dose-response curve (AUC) was determined for each drug and cell line using GraphPad Prism 5/8 (RRID: SCR_002798). The screened drugs were subsequently ranked according to their mean response (log [mean AUC]) in 1889c and MP57 (Table S1).

### Isolation of single cells from primary tissue

Single cells from primary tissues were isolated as previously described [17]. In brief, primary TH tissue samples were minced and washed with Organoid Wash Medium (OWM) containing advanced DMEM/F12, 10 mM HEPES, GlutaMAX, 100 μg/ml Primocin (Thermo Fisher, USA), and 0.1% bovine serum albumin (Sigma-Aldrich, USA). The samples were digested with Organoid Digestion Medium containing OWM with 0.1% Collagenase Crude Type XI (Sigma-Aldrich), 10.5 μM Y-27632 (AdooQ Bioscience, USA) and 10 μg/ml DNAseI (Sigma-Aldrich, USA). The cell suspensions were filtrated through a 100-micrometer mesh to remove residual tissue and cell agglomerates. The erythrocytes were lysed using the ACK Lysing Buffer (Gibco, USA). The cells were then resuspended in RPMI-1640 cell culture medium.

### Dynamic BH3 profiling

Dynamic BH3 profiling was performed as previously described [18] in technical triplicates. The cells were exposed to 12.5 nM carfilzomib for six hours before BH3 profiling. The cells were treated in a 384-well-plate with BH3 peptides using BIM at 10, 1, 0.3, 0.1, and 0.01 µM, BAD and HRK at 80 and 8 µM, and MS1 at 10, 3, and 1 µM. Treatment with the BIM peptide serves to assess the functionality of effector pro-apoptotic proteins and BAK. BAD selectively antagonizes the anti-apoptotic proteins BCL-2 and BCL-xL, while HRK and MS1 selectively antagonize the anti-apoptotic proteins BCL-xL and MCL-1, respectively [19]. Dimethyl sulfoxide (DMSO) was used as a negative control, and alamethicin (Ala) was used as a positive control. Intracellular cytochrome c was stained with an immunofluorescence-labeled antibody (clone 6H2.B4) (Biolegend, USA), and cells were subjected to flow cytometry. The relative cytochrome c release of E-cadherin-positive cells was evaluated using the following formula: 1 − [(sample-pos.ctrl.)/(neg.ctrl.-pos.ctrl.)].

### Proteasome activity-based fluorescent probe detection

The activity-based probe cocktail (ABP) and the 1 μM MVB003 (epoxyketone-based pan-reactive probe) were kindly provided by Florea et al. from Leiden University, The Netherlands [20]. Briefly, cells or five 10 µm fresh-frozen human tissue slices were harvested in M-PER buffer supplemented with 5 mM MgCl_2_ and 2 mM ATP, incubated on ice for 20 min, and purified by centrifugation at 14’000 rpm at 4°C for 25 min. Proteins were quantified using the Pierce 660 nm Protein Assay Reagent (Thermo) according to the Pierce Bovine Serum Albumin Standard Pre-Diluted Set (Thermo). The appropriate amount of premixed 10x ABP cocktail was mixed with 15 µg protein and then incubated at 37°C, 50 rpm in the dark for 1 hour. Proteins were separated on precast 12% Bis-Tris SurePage gels, and the gel was transferred to a translucent foil. Fluorescent bands were imaged in the wet gel slab using a BioRad Universal Hood III system using the Cy2, Cy3, and Cy5 fluorescent channels with an exposure time of 15 seconds for each wavelength.

For live cell inhibition, cell and tissue lysates were incubated with 12.5 nM carfilzomib for 30 minutes before incubation with 1 μM MVB003 for 1 hour at 37°C in a 5% CO2 humidified environment. Signals were detected using DyLight 550 (15 s exposure). Experiments were performed as biological triplicates.

### Cell viability assay and IC50 generation

Twenty-four hours after seeding (5000 cells per well) into 96-well plates, cells were incubated with treatment drugs at 1 to 1000 nM for an additional 72 h (Table S3). Cell viability was then determined using the CellTiter Glo One Solution Assay (Promega, USA). Relative IC50 was generated using Prism 8 (GraphPad Software, LCC) by normalizing individual measurements, log-transforming the drug concentration, and fitting a dose-response curve. Non-linear regression algorithms were used to calculate IC50 values from at least three biological replicates.

### Statistical analysis and data presentation

Statistical analyses were performed using Prism 10 (GraphPad Software, LCC). Optimal cut-off levels for clinical characteristics were calculated using the Cutoff Finder (https://molpathoheidelberg.shinyapps.io/CutoffFinder_v1). Kaplan-Meier curves were compared using the log-rank test. Significant differences between the two groups were calculated using Student’s t-test. Multiple t-tests were performed using one-way ANOVA. Bivariate correlations were carried out using Pearson (for continuous variables) or Spearman (for ordinal variables) correlation coefficients. Data are presented as mean ± standard error of the mean (SEM) unless stated otherwise. A significance was assumed at p-values *p* < 0.05.

p-values were annotated as follows: *p < 0.05; **p < 0.01; ***p < 0.001.

## Results

### Drug screening identifies specific vulnerabilities of 1889c and MP57 cells to the UPS inhibitors carfilzomib, TAK-243, and pevonedistat

**Fig. 1:**
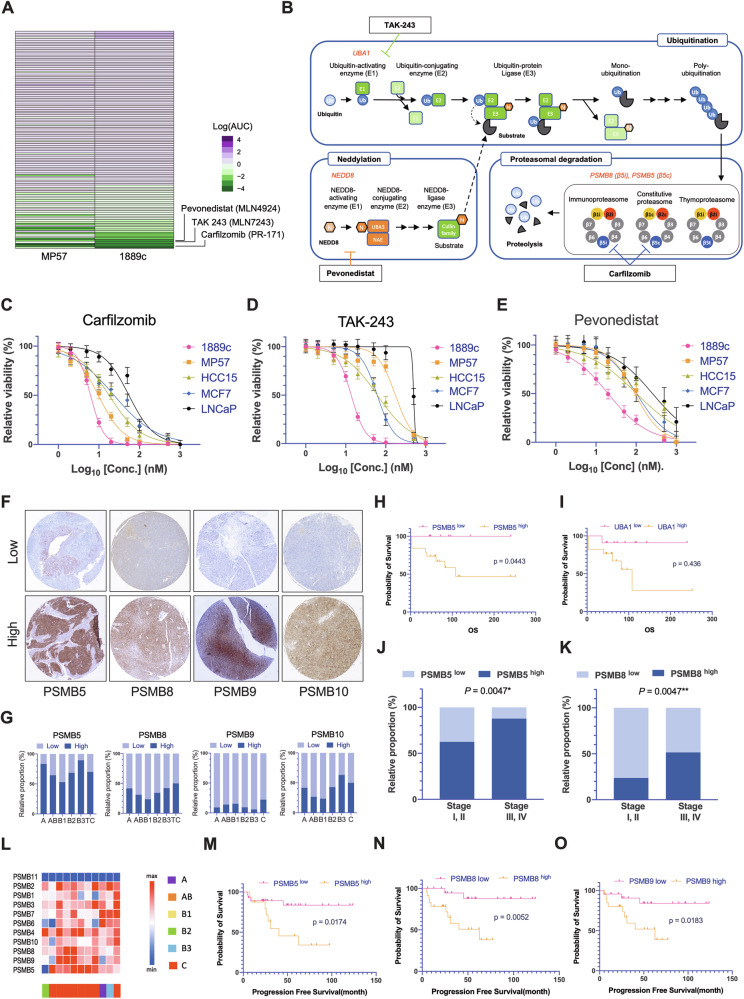
Clinical relevance and high expression of CPS and IPS make TC vulnerable to UPS inhibitors. **A** Color-coded response plot (log (AUC)) of the drug screening (*n* = 120) showing the UPS inhibitors carfilzomib, TAK-243, and pevonedistat as the most effective drugs against MP57 and 1889c. **B** Schematic representation of the ubiquitination, neddylation, and proteasomal degradation by carfilzomib, TAK-243, and pevonedistat. Affected genes are highlighted in red (PSMB5, PSMB8, UBA1, NEDD8). **C**–**E** Dose-response curve of the cell lines 1889c, MP57, HCC15, MCF7, and LNCaP treated with carfilzomib, TAK-243, and pevonedistat (0.1–1000 nM) treatment for 72 h. **F** Exemplary “high” and “low” IHC staining of TMA spots with antibodies against PSMB5, PSMB8, PSMB9, and PSMB10 and (**G**) the distribution within TH subtypes and TC. **H**, **I** Kaplan-Meier curves with the significant OS of PSMB5 (*p* = 0.0443) and UBA1 (*p* = 0.0436) among 28 B2, B3 TH and TC patients. **J** Significant increase of PSMB5 (*p* = 0.0047) and **K** PSMB8 (*p* = 0.0047) expression in Masaoka-Koga stages III and IV compared to stages I and II. **L** Shows a section (TC cluster) of a unsupervised hierarchical clustering of proteasome subunit expression (TCGA data) of 114 TH subtypes and TC (shown in Fig. S7). **L**–**O** Kaplan-Meier curves of B2 and B3 TH and TC TCGA expression data (*n* = 28) showing a significant difference in PFS of PSMB5 (*p* = 0.0174), PSMB8 (*p* = 0.0052) and PSMB9 (*p* = 0.0183).

To identify vulnerabilities of the TC cell lines 1889c [21] and MP57 (Figure. S1), we performed an unbiased pharmacological screening with a library containing 120 cytotoxic drugs (Fig. 1A). The UPS inhibitors carfilzomib (PR-171), TAK-243 (MLN7243), and pevonedistat (MLN4924) were among the top 10 compounds (Table S1**)**. Carfilzomib is a second-generation selective proteasome inhibitor [22] that covalently binds to the chymotrypsin-like 20S proteasome. It was approved by the FDA in 2012 for treating patients with refractory multiple myeloma [23]. The selective ubiquitin-activating enzyme, UAE (UBA1) inhibitor TAK-243 has been proposed as a potential therapeutic strategy for small cell lung cancer [24], and pevonedistat, a NEDD8 inhibitor, has been shown to have a significant therapeutic effect against relapsed/refractory multiple myeloma or lymphoma [25], metastatic melanoma [26], and advanced solid tumors [27]. Fig. 1B schematically shows the interference of carfilzomib, TAK-243, and pevonedistat with the UPS.

We compared the effects of carfilzomib, TAK-243, and pevonedistat in 1889c and MP57 with three solid tumor cell lines: the breast cancer cell line MCF7, the non-small cell lung cancer cell line HCC15, and the prostate cancer cell line LNCaP (Table S2).

Carfilzomib was the most potent drug tested, with IC50 values of 6.2 and 9.7 nM for 1889c and MP57, respectively. TAK-243 was active in 1889c with an IC50 of 13.7 nM but showed little activity in MP57 (IC50 of 175 nM). Pevonedistat had the lowest activity with the best potency in 1889c (IC50: 20.1 nM) (Fig. 1C–E and Table S3). We therefore decided to focus on proteasome inhibitors and did not further include TAK-243 and pevonedistat. We also tested the first-generation non-selective proteasome inhibitor bortezomib. 1889c and MP57 cells also showed the best response to bortezomib among the cell lines tested. However, the IC50 was 3-fold higher than carfilzomib’s (Fig. S1).

### UBA1, PSMB5, and PSMB8 protein expression in type B2 and B3 thymomas and thymic carcinomas has prognostic relevance

To evaluate the relevance of the UPS in TH and TC, we analyzed the expression of the proteasomal subunits β5c (PSMB5), β2c (PSMB7), β5i (PSMB8), β1i (PSMB9), β2i (PSMB10), β5t (PSMB11), UBA1, and NEDD8 by immunohistochemistry on tissue microarrays of 138 TET samples (Tables 1 and S4). Dividing the expression signals into high and low revealed a heterogeneous expression intensity of the UPS genes across all subtypes, with β5c (PSMB5) showing an increased expression in most patients (Fig. 1F and G). Tumors with high levels of PSMB7, PSMB9, and NEDD8 were observed in only a small fraction of patients, whereas UBA1, PSMB5, PSMB8, and PSMB10 were highly expressed in a significant percentage of cases (Figs. 1G and S2). As expected, the thymoproteasome subunit PSMB11 was present in B1, B2, and B3 TH and was absent in TC [12] (Fig. S2). To investigate the clinical role of the UPS in TH and TC, we correlated the immunohistochemical expression of the different subunits with the overall survival (OS), the progression-free survival (PFS), and the Masaoka-Koga stage in 28 clinical aggressive type B2 and B3 TH and in TC with known follow-up. High expression of PSMB5 and UBA1 was significantly associated with poor OS (Figs. 1H and S3), and PSMB5 and PSMB8 were enriched in tumors with Masaoka-Koga stage III and IV tumors compared to stage I and II tumors (Fig. 1I, J and S4).

### mRNA expression levels of UPS subunits show prognostic relevance in TH and TC

We next compared our immunohistochemical results with the expression of UPS genes in a TCGA dataset of *n* = 114 TET (Table S5). Hierarchical cluster analyses revealed high expression of UPS genes, including those of the immunoproteasome subunits in TC (Figs. 1L and S5). The UPS genes showed a positive correlation; thus, a specific correlation was observed by immunoproteasome subunits gene expression in TC (Fig. S6). High expression levels of PSMB5, PSMB8 and PSMB9 were highly significantly correlated with reduced PFS in B2, B3, TH and TC (Figs. 1 M–O and S7).

### Carfilzomib induces apoptosis in 1889c and MP57 cells

**Fig. 2:**
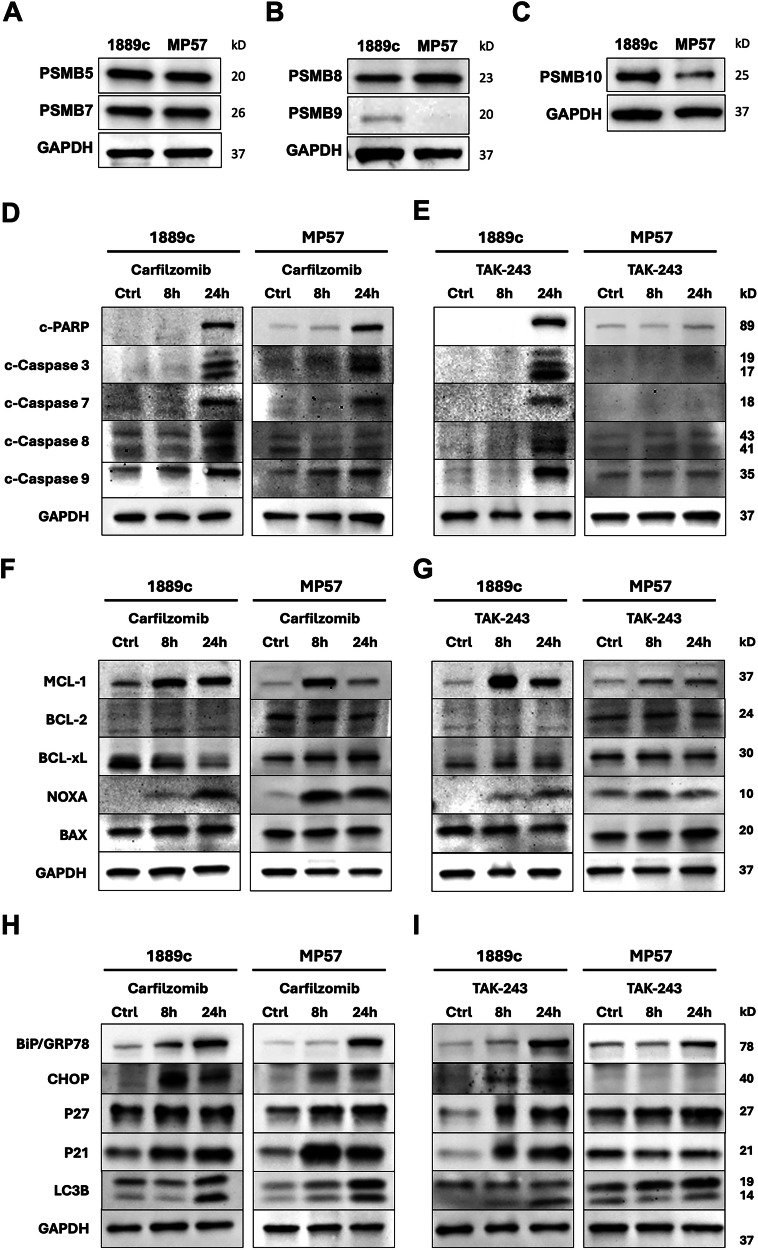
Carfilzomib and TAK-243 induce apoptosis, ER stress, and autophagy. **A**–**C** WB analysis of PSMB5, PSMB7, PSMB8, PSMB9, and PSMB10 showed a strong expression in MP57 and 1889c, except for PSMB9 in MP57. **D**, **E** Induction of apoptosis by PARP and caspase cleavage in 1889c and MP57 treated with carfilzomib (12.5 nM) and in 1889c treated with TAK-243 (12.5 nM) after 24 hours (**F** and **G**). Induction of BCL-2 family members after 8 and 24 hours treatment with carfilzomib and TAK-243. Carfilzomib (12.5 nM) transiently induced MCL-1 and NOXA after 8 hours and suppressed BCLxL signals and MCL-1 after 24 hours in 1889c cells. TAK-243 showed a strong induction of MCL-1 after 8 hours in 1889c but not in MP57. **H**, **I** Western blot analysis showed activation of cell cycle regulators (p27, p21), activation of the ER stress response (BiP/GRP, CHOP), and autophagy (LC3B) as early as 8 hours after treatment.

We next evaluated the expression of the proteasome subunits in the two TC cell lines1889c and MP57. Despite the apparent absence of PSMB9, both cell lines showed a strong protein expression of PSMB5 and PSMB7, and the immunoproteasome subunits PSMB8 and PSMB10 (Fig. 2A–C). Proteasome inhibition induces apoptosis in cancer [6, 28, 29], and we have recently described the role of the anti-apoptotic factors MCL-1 and BCL-xL in treatment resistance in TH and TC. Carfilzomib (12.5 nM) induced potent caspase- and PARP-dependent apoptosis in 1889c and MP57 within 24 hours. TAK-243 (12.5 nM), however, induced apoptosis only in 1889c but not in MP57 (Fig. 2E and F). Next, we tested the apoptosis regulatory factors BLC-2, MCL1, BCL-xl, BAX and NOXA. Carfilzomib treatment induced a strong MCL-1 expression in both cell lines as early as 8 hours after treatment. NOXA also increased over time and was accompanied by a decrease in MCL-1 levels at 24 hours (Fig. 2G and H). TAK-243 treatment showed a similar response in 1889c but did not affect MP57. Neither carfilzomib nor TAK-243 affected the expression of BCL-2 and BAX in the two cell lines.

Proteasome inhibition has also been shown to induce cellular stress through several other mechanisms, including cell cycle exit, ER stress, and autophagy [30–32]. Carfilzomib and TAK-243 treatment resulted in the accumulation and stabilization of the short-lived cell cycle regulatory proteins p21 and p27, the expansion of the ER stress response proteins BiP (GRP78) and CHOP, and the activation of autophagy by cleavage of LC3B [33] (Fig. 5I and J). We also tested bortezomib (100 nM) in the same setup in 1889c and MP57 which also induced apoptosis and showed a similar molecular response, also indicating a proteasome dependency of the two TC cell lines (Fig. S8A, B, and C).

The stability and autophagic degradation of the pro-apoptotic factor NOXA have been shown to play a critical role in bortezomib resistance [34]. We measured caspase activation in 1889c cells with siRNA knockdown of NOXA and treated with carfilzomib (Fig. S8D). NOXA knockdown samples did not show caspase activation, indicating that NOXA is essential for carfilzomib-induced apoptosis.

To test whether apoptosis is the primary cell death mechanism following proteasome inhibition by carfilzomib and TAK-243, we inhibited caspases by pre-treating the cells with zVAD. This completely rescued 1889c, confirming apoptosis as the responsible cell death signaling pathway (Fig. S8E).

### Low-dose carfilzomib treatment increases apoptotic priming in thymic carcinoma cells and synergizes with inhibitors of BCL2-family proteins

**Fig. 3:**
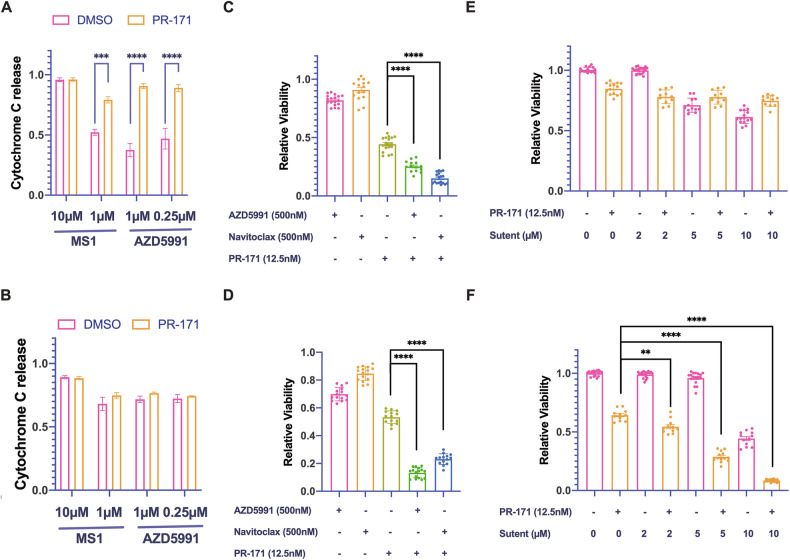
Dynamic BH3 profiling and combination treatment of 1889c and MP57 cells with carfilzomib, AZD5991, navitoclax and sunitinib. **A** Dynamic BH3 profiling showed a significant increase in apoptotic priming for MCL-1 after 6 hours of carfilzomib (12.5 nM) treatment in 1889c, **B** but not in MP57 cells. **C**, **D** Cell viability of 1889c and MP57 after 72 hours treatment with AZD5991 (500 nM), navitoclax (500 nM), carfilzomib (12.5 nM), and combined treatment with carfilzomib plus AZD5991 or navitoclax (p < 0.0001 compared to carfilzomib alone). **E** Combined treatment of 1889c and (**F**) MP57 cells with 12.5 nM carfilzomib and increasing concentrations (2–10µM) of sunitinib for 48 hours. Significant synergistic effects required high doses (2 µM) of sunitinib (*p* < 0.01).

Although proteasome inhibitors are successfully used in multiple myeloma, the induction of cellular resistance is a significant problem, and combination treatments are promising therapeutic approaches (reviewed in [35]). To explore similar strategies in TC, we performed dynamic BH3 profiling of 1889c and MP57 cells after pretreatment with sublethal concentrations carfilzomib (12.5 nM) for 6 hours. As described previously [17], we detected a baseline apoptotic priming for MCL-1 in 1889c cells that was even stronger for MP57. Carfilzomib pretreatment significantly increased cytochrome c release in 1889c cells treated with the MCL-1 specific peptide MS1 or the MCL-1 specific inhibitor AZD5991 (Fig. 3A), suggesting a potential synergy. In MP57 cells, BH3 profiling identified a strong functional dependence on MCL-1 (Fig. 3B). Strikingly, BCL2-inhibitor navitoclax and the MCL-1 inhibitor AZD5991 in combination with low-dose carfilzomib (12.5 nM) showed strong synergistic effects in both cell lines (Fig. 3C and D). To validate these synergies, we calculated synergy scores using SynergyFinder 2.0. 1889c showed a strong synergy with carfilzomib and AZD5991 (Fig. S9A) and MP57 with carfilzomib and navitoclax (Fig. S9B) indicating different but specific vulnerabilities. FACS analysis using PI and annexin V stainings as well as immunoblot analyses of cleaved PARP and caspase 3 in cells treated with corresponding combinations revealed a massive induction of apoptosis (Figs. S9 and S10). In contrast, the same treatment showed a much less pronounced effect in MCF7 and LNCaP cells (Fig. S9B), suggesting a prominent and tumor-specific role of the UPS in TC cells.

Receptor tyrosine kinase receptor inhibitors in combination with proteasome inhibitors have recently been proposed as a treatment option in multiple myeloma [36]. Sunitinib is the only clinically approved second-line therapy in relapsed TH and TC [37]. Therefore, we tested the combination of carfilzomib plus sunitinib and detected a synergistic effect in MP57 but not in 1889c cells (Fig. 3E and F).

### The expression level of immunoproteasome subunits in TET tissues and cancer cell lines determines response to carfilzomib

**Fig. 4:**
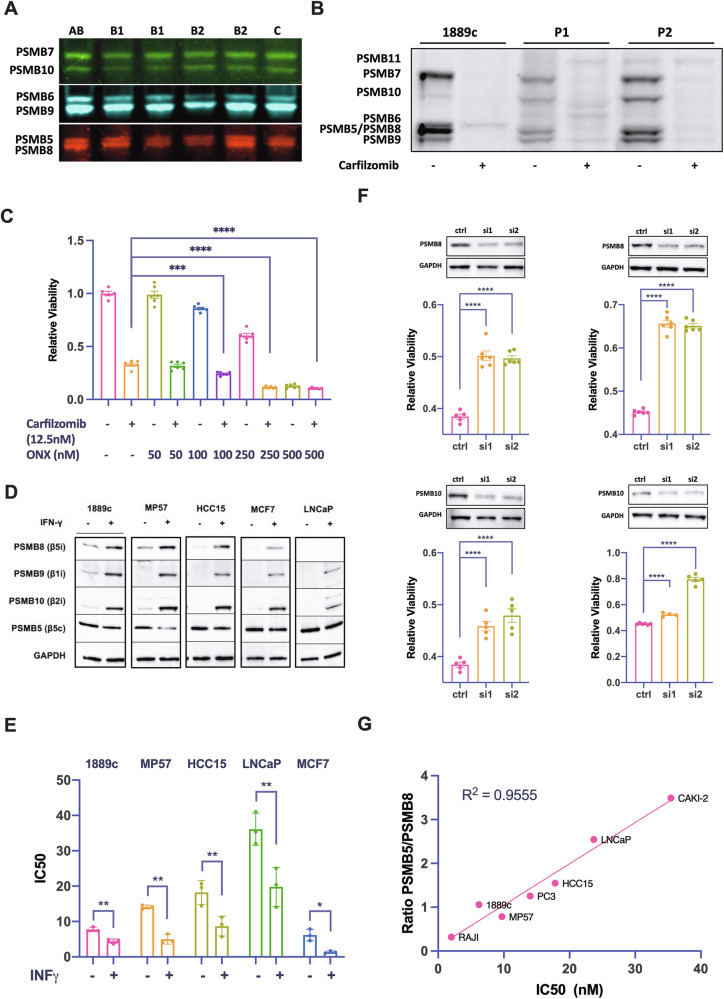
Proteasome activity and inhibition in cell lines and TET tissue and the relevance of PSMB8 in carfilzomib treatment. **A** ABP fluorescent labeled constitutive and immunoproteasome subunit activity in protein lysates of TET showing a strong immunoproteasome activity in TET. **B** The addition of carfilzomib to lysates of 1889c cells and two TET patient tissues samples (P1 (type B3) and P2 (type B2)) resulted in a strong inhibition of the constitutive and the immunoproteasome. **C** A single treatment with increasing concentration of immunoproteasome specific inhibitor ONX (50 - 250 nM) or combined with a sublethal concentration of carfilzomib (12.5 nM) of 1889c over 48 hours. **D** IFN-γ (100 IU/ml, 48 hours) induced immunoproteasome subunits in 1889c, MP57, HCC15, MCF7, and LNCaP and (**E**) significantly increased the response to carfilzomib. **F** Knockdown of PSMB8 and PSMB10 with two specific siRNAs (si1, si2) in 1889c (upper and lower panels left) and MP57 (upper and lower panels right) significantly decreased the response to carfilzomib. **G** Strong correlation between the PSMB5:PSMB8 protein expression ratio and the response to carfilzomib in Raji, 1889c, MP57, PC3, HCC15, LNCaP, and CAKI2 cells (R^2^ = 0.9555, p < 0.0001). **p < 0.01; ***p < 0.001; ****p < *0.0001*.

The high abundance of immunoproteasome subunits in TC tissues and cell lines suggested an association with the response to carfilzomib. We first tested the activity of different proteasome subunits using an activity-based probe (ABP) cocktail in tissue lysates from 5 TH and 1 TC [20]. This revealed a strong activity of the immunoproteasome subunits β1i (PSMB9), β2i (PSMB10), and β5i (PSMB8) in all samples (Fig. 4A). Addition of carfilzomib to TH tissue lysates and 1889c cells resulted in a broad inhibition of constitutive and immunoproteasome subunits. The activity of the thymoproteasome subunit PSMB11 remained unaffected (Fig. 4B). To further analyze the specific importance of the immunoproteasome for the carfilzomib response, we treated 1889c cells with low doses of the β5i (PSMB8) inhibitor ONX but found only minor effects on cell viability. Only high concentrations of ONX (250 and 500 nM) significantly reduced cell viability (Fig. 4C).

Next, we treated 1889c, MP57, HCC15, and LNCaP cells with interferon-gamma (INFg) to induce immunoproteasome expression. All treated cell lines showed increased expression of the immunoproteasome subunits β1i (PSMB9), β2i (PSMB10), and β5i (PSMB8) (Fig. 4D) and a significantly increased response to carfilzomib (Fig. 4E). Conversely, siRNA knockdown of the immunoproteasome subunits β5i (PSMB8) and β2i (PSMB10) dramatically reduced the response to carfilzomib in 1889c and MP57 cells (Fig. 4F). The IFNg-induced phenotype could be recapitulated by transient overexpression of β5i (PSMB8) (Fig. S11). Finally, there was a strong and statistically highly significant correlation between the ratio of the CPS subunit β5 (PSMB5) to the IPS subunit β5i (PSMB8) and the response to carfilzomib across a spectrum of seven cancer cell lines (Fig. 4E), indicating that the higher the expression of the β5i (PSMB8) subunit, the better the response to carfilzomib in a given tumor.

### High PSMB8 expression distinguishes TSCC from other squamous cell carcinomas

**Fig. 5:**
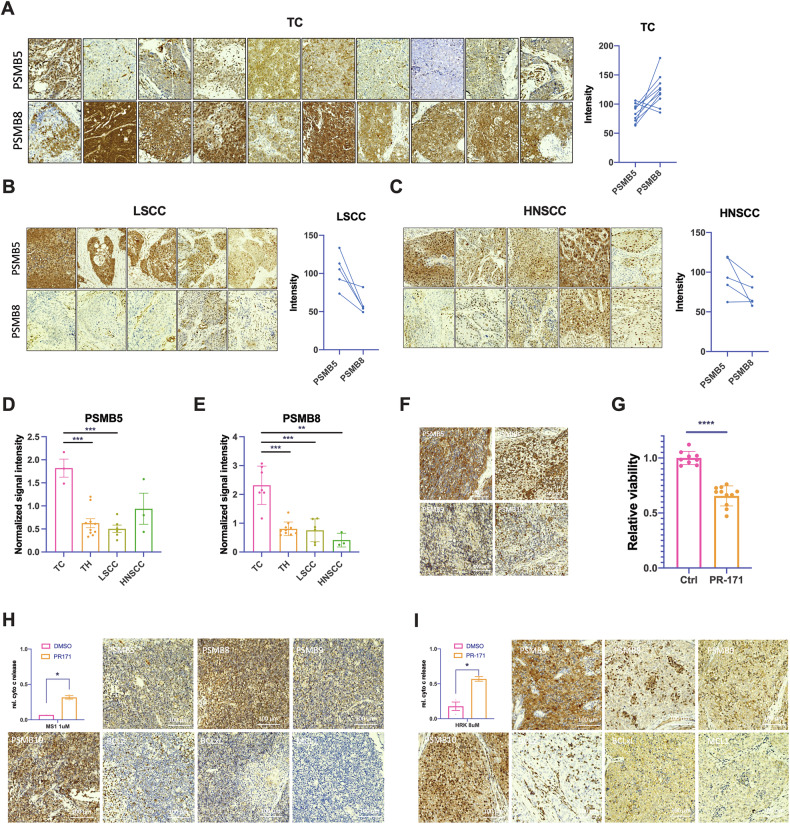
High expression of PSMB8 is a specific feature of thymic carcinomas compared to cancers of the lung and head and neck. **A** Higher immunohistochemical expression of PSMB8 than PSMB5 in 10 TC compared to (**B**) 5 LSCC and (**C**) 5 HNSCC. **D** Significantly higher protein levels of PSMB5 in 3 TC vs. 10 TH, 6 LSCC, and 3 HNSCC and (**E**) significantly higher protein levels of PSMB8 in 6 TC vs. 10 TH, 6 LSCC, and 3 HNSCC. **F** Immunohistochemical expression of PSMB5, PSMB8, PSMB9, and PSMB10 in a type B3 thymoma and (**G**) significant response of the primary cells of the same tumor to carfilzomib (PR-171) (12.5 nM for 72 h). **H**, **I** Dynamic BH3 profiling of primary cells treated with 12.5 nM carfilzomib (PR-171) for 6 h and IHC staining for PSMB5, PSMB8, PSMB9, PSMB10, BCL2, BCL-xL, and MCL-1 in two TH patient tissue samples. *p < 0.05; **p < 0.01; ***p < *0.001*.

To determine whether thymic carcinomas are particularly amenable to carfilzomib treatment compared to similar tumors from other organs, we analyzed the expression of the CPS subunit β5 (PSMB5) and the IPS subunit β5i (PSMB8) in tissue samples of *n* = 10 TC and *n* = 5 lung squamous cell carcinomas (LSCC) and *n* = 5 head and neck squamous cell carcinomas (HNSCC) by immunohistochemistry (Fig. 5A-C) and western blotting (Fig. 5D and E). This revealed an increased expression of the IPS subunit β5i (PSMB8) in most TC but not in LSCC and HNSCC (Fig. 5D and E). A primary ex vivo short-term culture of a type B2 TH showed a significant decrease in cell viability after treatment with 100 nM carfilzomib at 48 h (Fig. 5F). In addition, dynamic BH3 profiling of two fresh primary TH samples showed a significant increase in apoptotic priming for MCL-1 and BCL-xL after 6 hours of carfilzomib treatment (Fig. 5G and H), indicating that not only TC but also TH patients may be promising candidates for carfilzomib therapy.

## Discussion

Treatment options for aggressive TET are currently limited. Most TH and TC do not harbor recurrent oncogenic driver mutations, thus making the identification and therapeutic exploitation of non-mutational mechanisms a top priority [38]. Thymic epithelial cells are professional antigen-presenting cells and the only epithelial cell type in the human body that expresses all three major proteasome subtypes: the constitutive proteasome, the immunoproteasome, and the thymoproteasome.

However, the expression and function of the different proteasome classes in TET have not been investigated. We hypothesized that TET may be ideal candidates for treatment with proteasome inhibitors, a group of compounds typically used to treat multiple myelomas, but not usually used for solid tumors.

In an unbiased in vitro drug screen of 120 cytotoxic drugs, the two thymic carcinoma cell lines 1889c and MP57 showed exquisite sensitivity to the UPS inhibitors carfilzomib (PR-171), TAK-243 (MLN7243), and pevonedistat (MLN4924). Carfilzomib, a second-generation proteasome inhibitor, induced ER stress and autophagy followed by apoptosis in 1889c and MP57 at significantly lower concentrations than in the non-TET cell lines HCC15, MCF7, and LNCaP. Carfilzomib has been successfully used in the treatment of multiple myeloma, increasing the overall response rate (ORR) by 10.4% and the overall survival OS by 8.3% compared to the control group [39, 40]. The IC50 of carfilzomib in 1889c and MP57 was comparable to published data using carfilzomib in multiple myeloma cell lines [41]. Carfilzomib irreversibly binds to the CPS subunit PSMB5 of the 20S proteasome but also efficiently inhibits the immunoproteasome subunit β5i (PSMB8) [42]. Cells with high immunoproteasome expression are generally more sensitive to proteasome inhibition [43, 44]. Niewerth et al. described that PSMB8 induced by INF-γ re-sensitized bortezomib-resistant hematological cancer cells [45]. Here, we provide experimental evidence that these general mechanisms also apply to thymic carcinomas: up to 50% of thymic carcinomas showed strong immunohistochemical expression of immunoproteasome subunits: i) epithelial cells of thymomas and thymic carcinomas showed functional activity of the immunoproteasome subunits β1i (PSMB9), β2i (PSMB10), and β5i (PSMB8). ii) Treatment of several cancer cell lines including TC with INF-γ induced the immunoproteasome and rendered the cells more susceptible to carfilzomib. Conversely, siRNA knockdown of the immunoproteasome subunits β5i (PSMB8) and β2i (PSMB10) dramatically reduced the response to carfilzomib in 1889c and MP57 cells. iii) There was a strong and statistically significant correlation between the expression level of the immunoproteasome subunit β5i (PSMB8) and the response to carfilzomib among the cancer cell lines tested. Furthermore, we were able to show that TC are distinguished from histologically similar squamous cell carcinomas in other organs (e.g., lung and head and neck) by their increased expression of the immunoproteasome β5i (PSMB8) subunit together with high levels of the CPS subunit β5 (PSMB5). Taken together, these data strongly suggest that TC are ideal candidates for targeted treatment with proteasome inhibitors such as carfilzomib. There is even circumstantial clinical evidence to support this hypothesis: a case report described a 70-year-old patient with stage III TC who had a complete response and remained disease-free for four years after local radiotherapy. At the same time, however, the patient was receiving bortezomib for multiple myeloma [46]. The authors stated that they could not exclude that the patient’s outcome was influenced by bortezomib. The relative toxicity of proteasome inhibitors and acquired resistance is a major concern [47, 48]. In addition, the short pharmacologic half-life and poor tissue penetration of carfilzomib in vivo are major obstacles in the treatment of solid tumors [49, 50]. Carfilzomib and bortezomib are not the only clinically approved proteasome inhibitors. The FDA-approved proteasome inhibitor ixazomib (Ninlaro) is also used to treat multiple myeloma and has the advantage of being orally administered. Ixazomib has been shown to efficiently penetrate solid tumors and inhibit the growth of pulmonary osteosarcoma metastases in mice [51]. Toxicity and the development of resistance will remain major obstacles to the translation of proteasome inhibitors into the treatment of TET patients. However, as a proof of concept, we were able to show here that low-dose carfilzomib increases apoptotic priming of TC cells and synergizes with BCL2 family protein inhibitors such as navitoclax or AZD5991, suggesting that appropriate synergistic drug combinations could be used to lower the doses of individual drugs as a way to minimize toxicity without losing efficacy. Finally, although this study was designed to explore the potential use of proteasome inhibitors in TC, initial in situ and in vitro data suggest that the same strategy may also work in thymoma. Thymoma tissue samples also showed high expression levels of CPS and IPS subunits in a percentage of cases and functional activity of both proteasome classes in vitro. In addition, short-term epithelial cell cultures of two thymoma samples showed significant therapeutic effects after treatment with carfilzomib in vitro. We have previously reported on upfront BH3 profiling of fresh TET samples to tailor therapy with BCL2 protein inhibitors [17]. We have shown here that the same method can be used to extend this screening to select optimal candidates for treatment with proteasome inhibitors or even a combination of both.

## Supplementary information

Supplementary Figures

Table S1

Table S2

Table S3

Table S4

Table S5

Table S6

Table S7

Original Western Blots

## Data Availability

The datasets supporting the conclusions of this article are included within the article and its supplementary files.
