# Supplementary material for: Proteasome inhibition as a potential therapeutic target in thymic cancer
Source: Cell Death Dis. 2025 Dec 4;16(1):885. doi: 10.1038/s41419-025-08240-5 (PMC12698856; doi:10.1038/s41419-025-08240-5)
Supplement: Supplementary file 1 — Supplementary Figures [file 41419_2025_8240_MOESM1_ESM.doc]

**Supplementary Methods and Figures**

**Proteasome inhibition as a potential therapeutic target in thymic cancer**

Satoru Okada^1,2^, Louisa Benter^1^, Leon Schrell^1^, Denise Müller^1^, Selen Selcen^1^, Hanibal Bohnenberger^1,4^, Carolin Schneider^3^, Günter Schneider^3,4^, Melanie Lohrberg^5^, Raphael Koch^5,4^, Tobias R. Overbeck^6,4^, Alexander von Hammerstein-Equord^7^, Stefan Welter^8^, Marc Hinterthaner^7^, Lucia Cordes^8^, Katayoon Shirneshan^4^, Christoph Netzer^9,10^, Masayoshi Inoue^2^, Alexander Marx^1^, Philipp Ströbel^1,4, #^, Stefan Küffer^1^

**Supplementary Methods**

***Immunohistochemistry***

Tissue microarrays containing two 1.5mm representative punches of TET samples were used for IHC (**Table 1**). The proteasome subunits β5c (*PSMB5*), β2c (*PSMB7*), β5i (*PSMB8*), β1i (PSMB9), β2i (PSMB10), β5t (*PSMB11*), ubiquitin-activating enzyme (*UBA1*), and NEDD8 (*NEDD8*) were evaluated. Staining was performed on 2-µm sections according to a standard protocol on an Autostainer (Dako Agilent, USA). In brief, antigen retrieval was performed at 95°C in pH 6 or pH 9 Envision FLEX target retrieval solution in a PT Link Module (Agilent, USA) followed by 1h incubation with primary antibodies (**Table S6**). Samples were washed with PBS and incubated with an appropriate secondary antibody (EnVision Flex+, Dako) for 30 min. Staining was evaluated for two cores of a respective case. Signal intensities were judged from 0 – 2 per core, and the average signal was classified into “Low” < 1 or “High” ≥ 1.

***Analysis of publicly available TCGA data sets***

We analyzed the TET dataset (annotated as THYM) of TCGA (CBioPortal, <http://www.cbioportal.org/>) [1], which was curated according to Radovich et al. [2]. To make consistent comparisons with our cohort, we excluded neuroendocrine thymic carcinomas (n=1) and micronodular thymomas (n=2) and analyzed the TCGA TET cohort (n=114) that comprised type A (n=10), AB (n=48), B1 (n=12), B2 (n=25), B3 (n=10), and TCs (n=10) (**Table S5**). z-Scores were extracted for the respective genes and optimal signal cut off for Kaplan Meier curves and High and Low analyses were calculated using Cutoff Finder (https://molpathoheidelberg.shinyapps.io/CutoffFinder_v1/ ).

***siRNA transfection and vector transfection***

For siRNA cell transfection, we used the Lipofectamine RNAiMAX transfection reagent (Thermo Fisher, USA) according to the manufacturer’s protocol. Briefly, 250μl serum-free RPMI-1640 transfection mix, including 100 nM siRNA **(Table S7)** and 7.5μl transfection reagent, was incubated for 5 min and added to 4 × 10^5^ cells and incubated for 24 hours.

For plasmid transfection, we used the Lipofectamine 3000 transfection reagent (Thermo Fisher, USA) according to the manufacturer’s protocol. Briefly, 250μl serum-free RPMI-1640 transfection mix, containing 2µg PSMB8 or vector control DNA **(Table S7),** was incubated for 20 min and added to 4 × 10^5^ cells. Gene knockdown and overexpression (OE) were validated by Western blots 24 hours after transfection. Timepoint 0 hours for drug treatments was defined as 24 hours after transfection, and for the time indicated.

| **Drug** | **IC50 (nM)** | | | | |
| --- | --- | --- | --- | --- | --- |
|  | **1889c** | **MP57** | **HCC15** | **MCF7** | **LNCaP** |
| **Carfilzomib (PR-171)** | 6.2 | 9.7 | 17.8 | 23.7 | 54.9 |
| **TAK-243 (MLN7243)** | 13.7 | 175 | 61.5 | 61.2 | 499 |
| **Pevonedistat (MLN4924)** | 20.1 | 92.9 | 103 | 102 | 256 |

**Table S3: IC50 calculation for carfilzomib, TAK-243, and pevonedistad in cell lines.** IC50 values of 1889c, MP57, HCC15, MCF7, and LNCaP for Carfilzomib**,** TAK-243, and Pevonedistad treatment for 72h.

**Figure S1. IC50 calculation of Bortezomib in different cancer cell lines.** Dose-response curve and IC50 values of 1889c, MP57, HCC15, MCF7, and LNCaP for 1^st^ generation non-selective proteasome inhibitor Bortezomib treated for 72h.

**Figure S2. Immunohistochemistry analysis of UPS-related protein expressions in 138 patients with thymic tumors.** “High” and “Low” IHC staining of PSMB7, PSMB11, UBE1, and NEDD8. Average staining intensities from duplicate cores on TMAs: 0 = neg, 1 = weak, 2 = strong. Definition of “High” and “Low”: Low < 1, High ≥1.

**Figure S3. Overall survival according to expression levels of UPS-related proteins in 28 patients with thymic tumors.** (A-E) Kaplan-Meier curves of OS according to expression levels of PSMB7, PSMB8, PSMB9, PSMB10, and NEDD8 and (F-K) of PFS survival of PSMB5, PSMB7, PSMB8, PSMB9, PSMB10, and UBE1 of 28 patients with aggressive B2 and B3 TH and TC. Cut off point was calculated using Cutoff Finder (https://molpathoheidelberg.shinyapps.io/CutoffFinder_v1)

**Figure S4. Immunohistochemical expression of UPS subunits (high and low, IHC) in correlation with Masaoka stages I and II vs. III and IV.**

**Figure S5. Proteasome subunit expression heatmap of 114 thymic tumors extracted from the TCGA database.** Unsupervised clustering of PSMB1- PSMB10 expression (z-Score) in 114 TET samples using MORPHEUS (https://software.broadinstitute.org/morpheus/).

**Figure S6. Proteasome subunit correlation of TCGA mRNA expression in 114 thymic tumors (covering all histological subtypes).** Correlation heatmaps were generated using GraphPad Prism (Version 10.3.1).

**Figure S7. Overall (OS) and progression-free survival (PFS) according to mRNA expression levels of UPS-related proteins in the TCGA cohort of thymic tumors.** (A-H) OS Kaplan-Meier curves with calculated optimal mRNA expression cut-off of PSMB5, PSMB6, PSMB7, PSMB8, PSMB9, PSMB10, UBA1and NEDD8 and (I-M) PFS of PSMB6, PSMB7, PSMB10, NEDD8, and UBA1 of 44 patients with aggressive B2 and B3 TH and TC.

**Figure S8. Western blot and functional analysis of bortezomib and apoptotic induction via Noxa upregulation**. (A) Western blot analysis showing PARP cleavage and caspase activation (both intrinsic and extrinsic pathway) of 1889c and MP57 after a 24 hours single treatment with Bortezomib (12.5 nM), indicating apoptosis induction. (B) Western blot analysis of BCL-2 family members after a 24 hours single treatment with Bortezomib (12.5 nM), showing common MCL-1 and Noxa upregulation in both cell lines. (C) Western blot analysis of ER stress (BiP/GRP78, CHOP), and autophagy (LC3B), cell cycle (p27, p21) markers after a 24 hours single treatment with Bortezomib (12.5 nM). (D) Western blot analysis showing that silencing NOXA by siRNA suppresses the activation of initiator of intrinsic pathway (Caspase 9) and executioner Caspase 3 and PARP in 1889c cells after a 24 hours single treatment with Carfilzomib (100 nM). (E) Cell viability assay of 1889c treated for 24h with Carfilzomib (100 nM), TAK-243 (100 nM), and staurosporine (0.25 μM) alone or after 3h pretreatment with the pan-caspase inhibitor zVAD-fmk (100 μM).

*****, p<0.0001. ER, endoplasmic reticulum.*

**Figure S9. Combined treatment of carfilzomib/TAK-243 and BH3 mimetics.**

(A) Dose-response matrix and IC50 values of 1889c for the best combination of carfilzomib (0–1000nM) and AZD5991 (0, 100, 500, 1000nM), and (B) those of MP57 for the best combination of carfilzomib (0–1000nM) and navitoclax (0, 100, 500, 1000nM) using SynergyFinder 2.0 [https://synergyfinder.fimm.fi](https://synergyfinder.fimm.fi/) .

**Figure S10. Powerful induction of apoptosis by a synergistic combination of carfilzomib/TAK-243 and BH3 mimetics.** FACS study using PI and Annexin V staining showing a strong induction of apoptosis (A) after 24 hours combined treatment of Carfilzomib (100nM) / TAK-243 (100nM) and AZD5991 (500nM) in 1889c, and (B) after 24 hours combined treatment of Carfilzomib (100nM) and Navitoclax (500nM) in MP57.

(C) PARP cleavage and Casp3 activation in 1889c and MP57 showing synergistic apoptosis induction by WB when treated with a combination of sublethal concentrations of carfilzomib (25nM) and AZD5991 (500nM) after 24 hours. **, p<0.05; **, p<0.01; ***, p<0.001; ****, p<0.0001.*

**Figure S11. Overexpression of PSMB8 in thymic carcinoma cell lines increases their vulnerability towards carfilzomib.** WB (A) of control vector (ctrl) and PSMB8 overexpression (OE) in TC cell lines. (B) Significant increased effect of carfilzomib in 1889c and MP57 overexpressing PSMB8.

References Supplementary Methods

1. Gao J, Aksoy BA, Dogrusoz U, Dresdner G, Gross B, Sumer SO, Sun Y, Jacobsen A, Sinha R, Larsson E, et al: **Integrative analysis of complex cancer genomics and clinical profiles using the cBioPortal.** *Sci Signal* 2013, **6:**pl1.

2. Radovich M, Pickering CR, Felau I, Ha G, Zhang H, Jo H, Hoadley KA, Anur P, Zhang J, McLellan M, et al: **The Integrated Genomic Landscape of Thymic Epithelial Tumors.** *Cancer Cell* 2018, **33:**244-258 e210.
