# Supplementary material for: Proteasome inhibition as a potential therapeutic target in thymic cancer
Source: Cell Death Dis. 2025 Dec 4;16(1):885. doi: 10.1038/s41419-025-08240-5 (PMC12698856; doi:10.1038/s41419-025-08240-5)

Figure 2a-d WB1

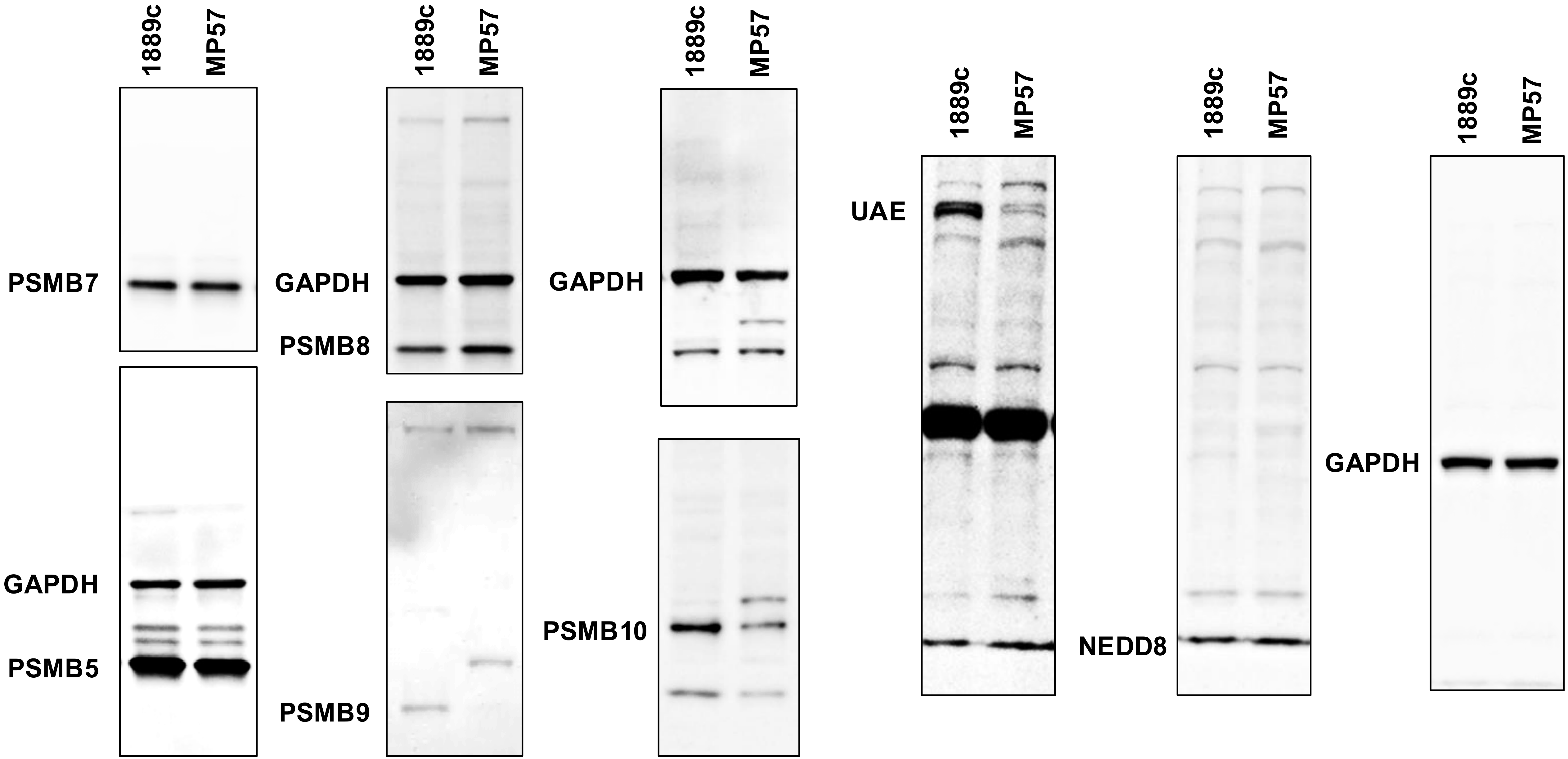

Figure 2e WB2

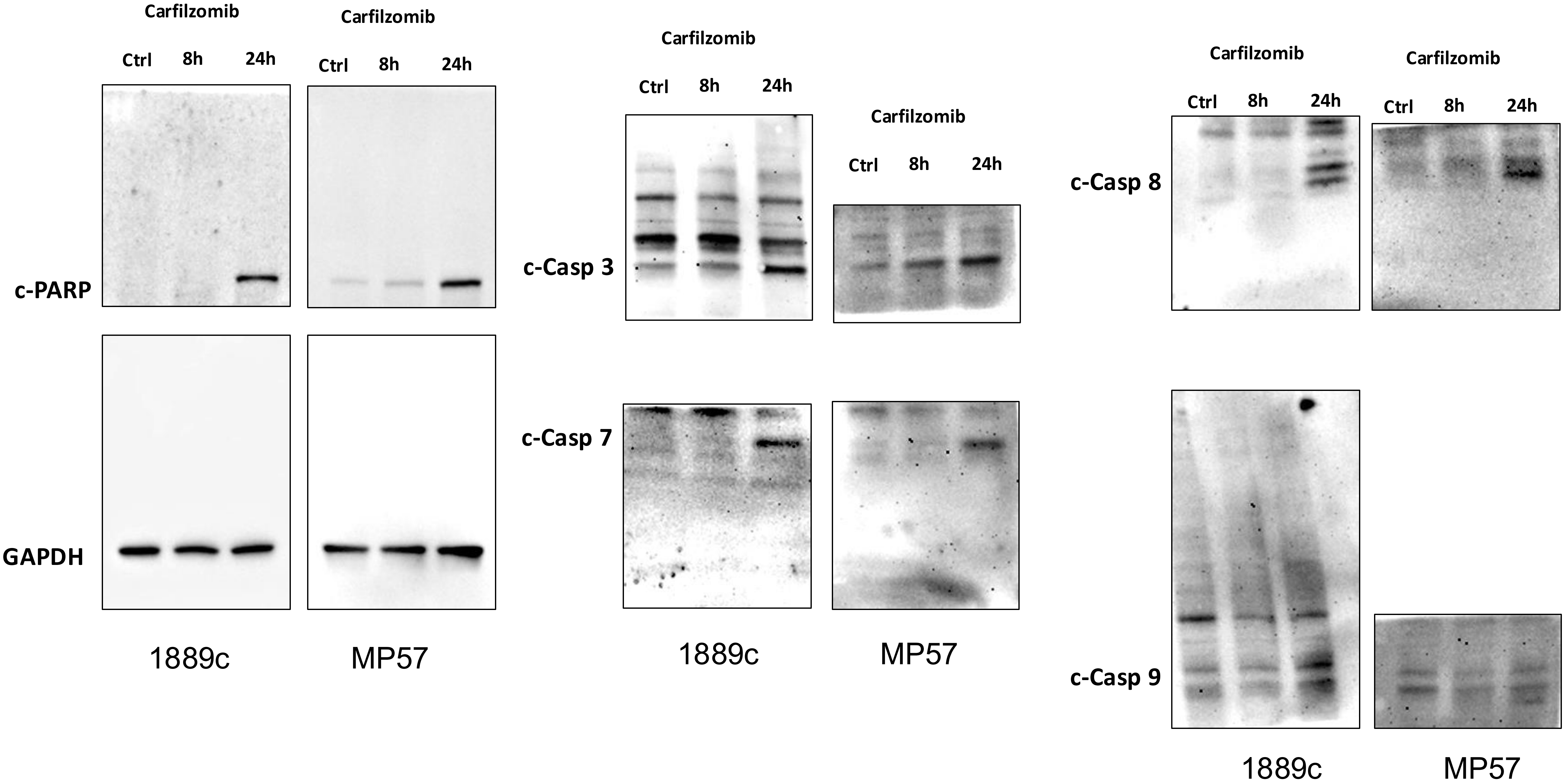

Figure 2f WB3

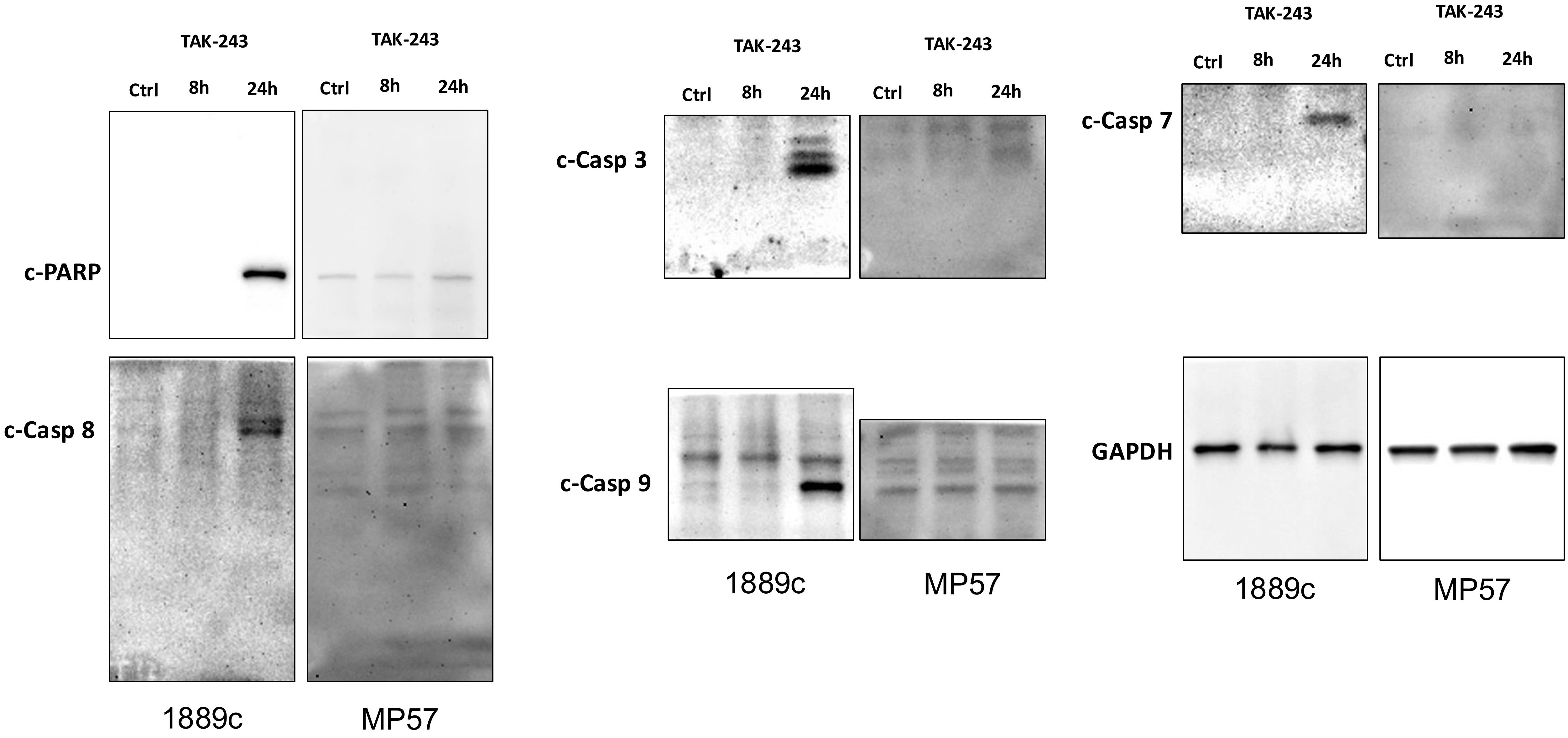

Figure 2g WB4

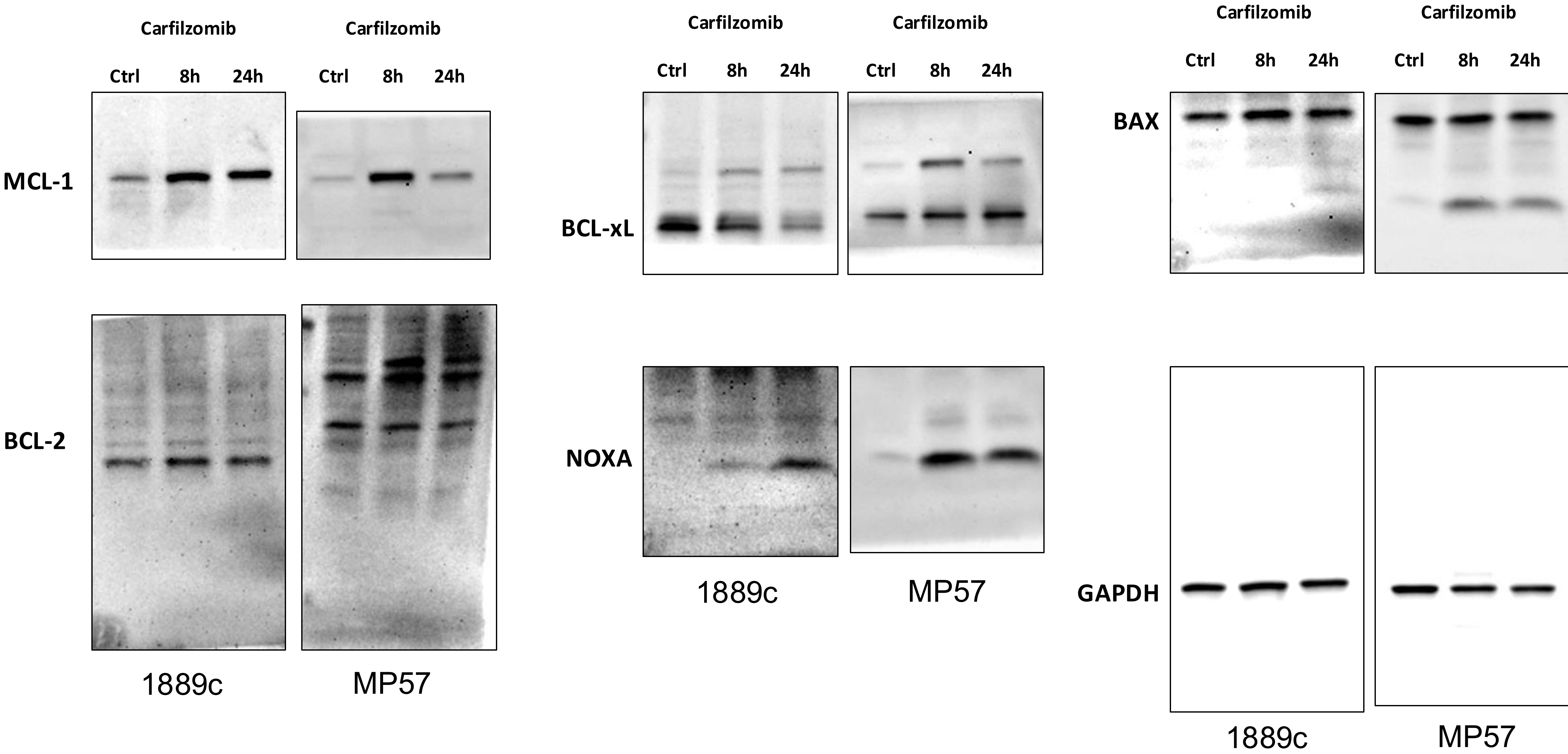

Figure 2h WB5

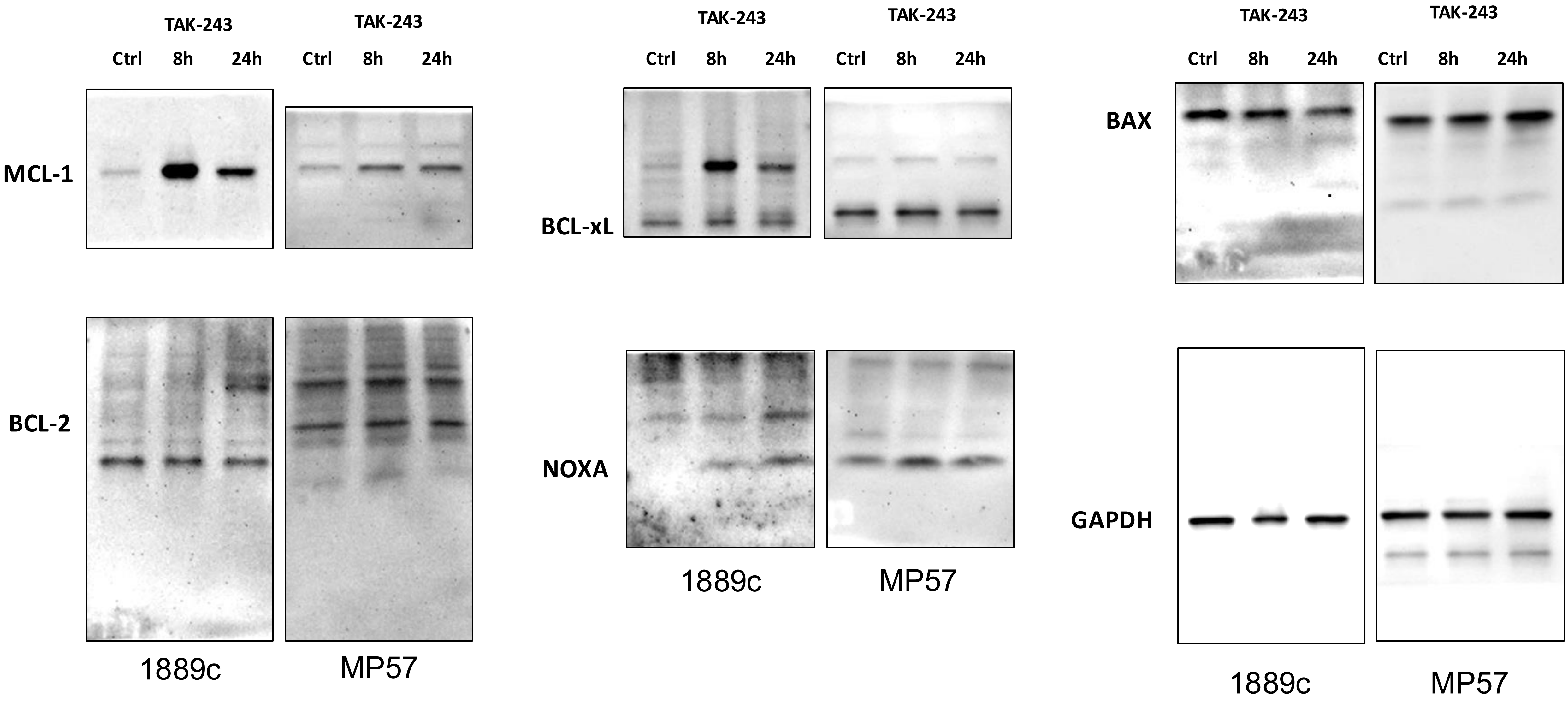

Figure 2h WB6

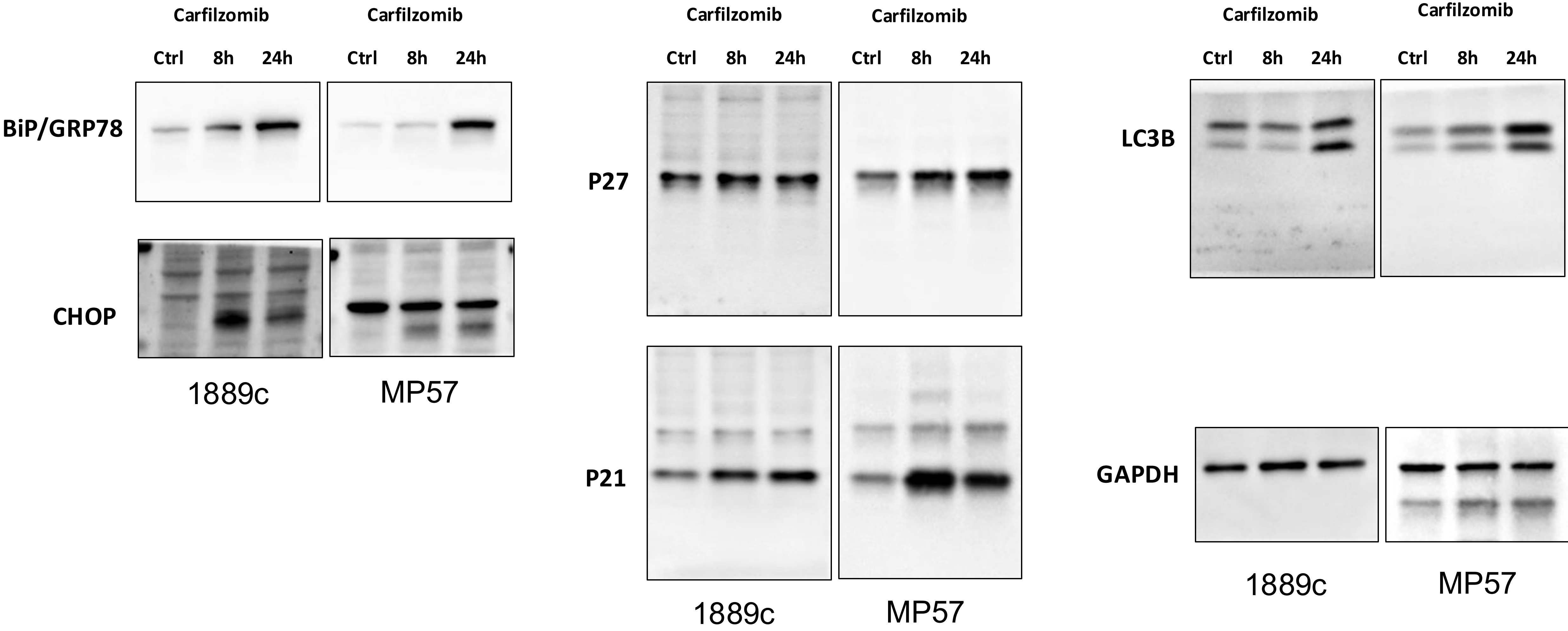

Figure 2h WB7

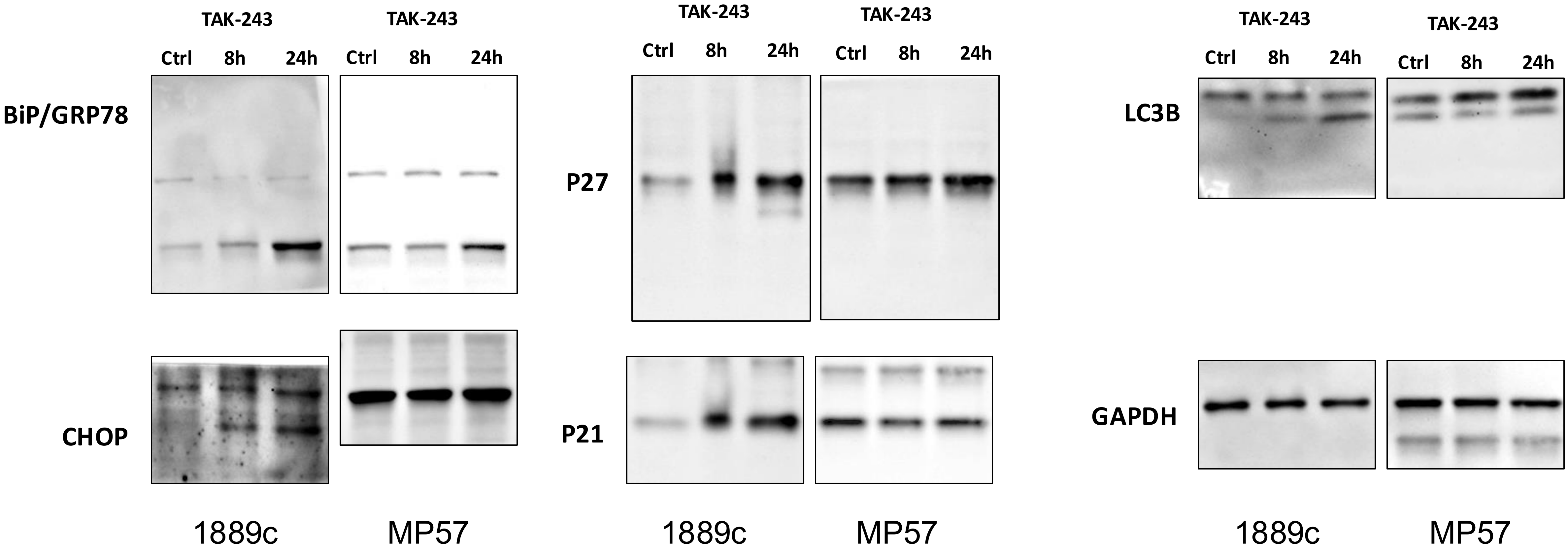

Figure 4A WB8

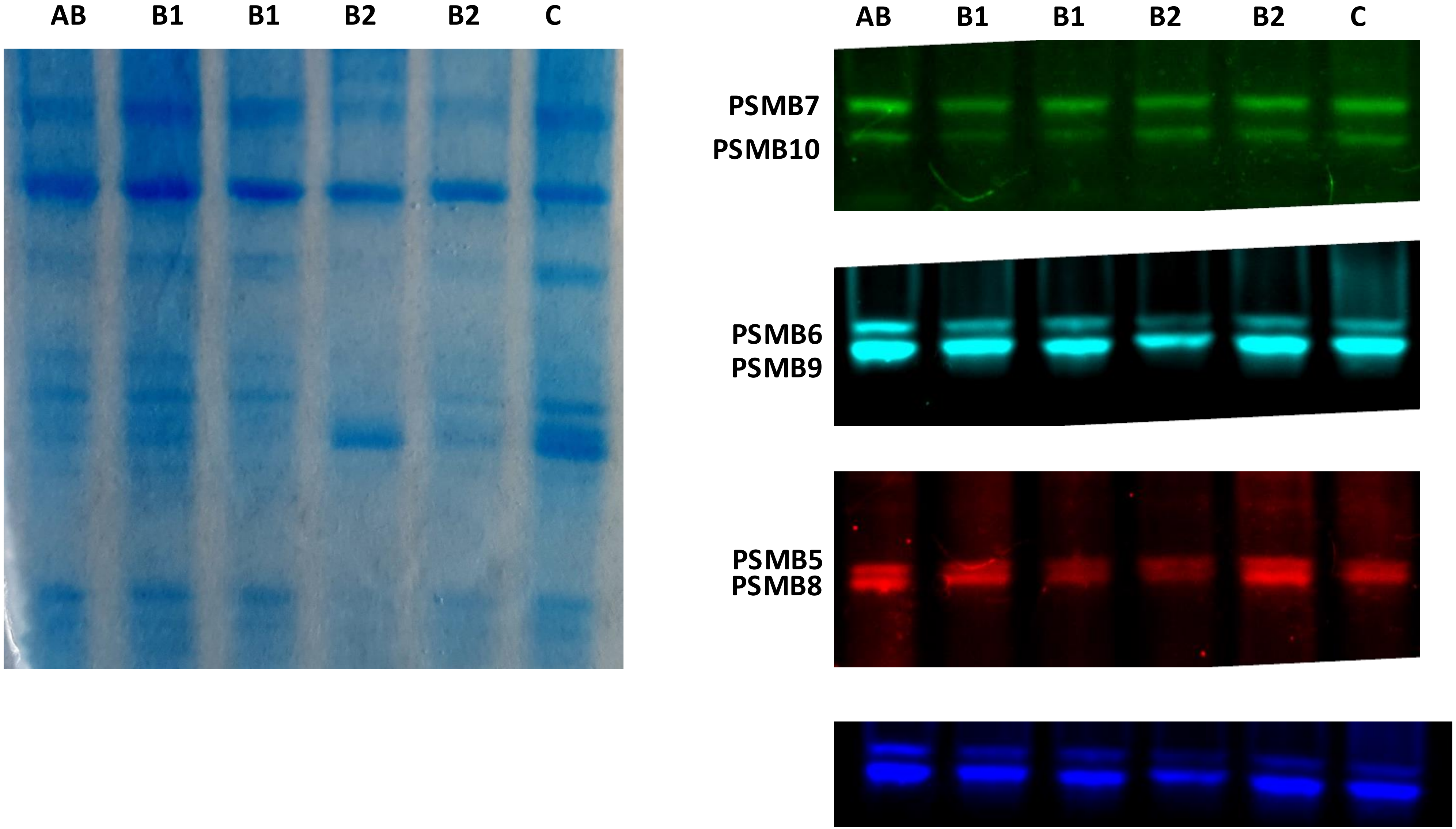

Figure 7f WB9

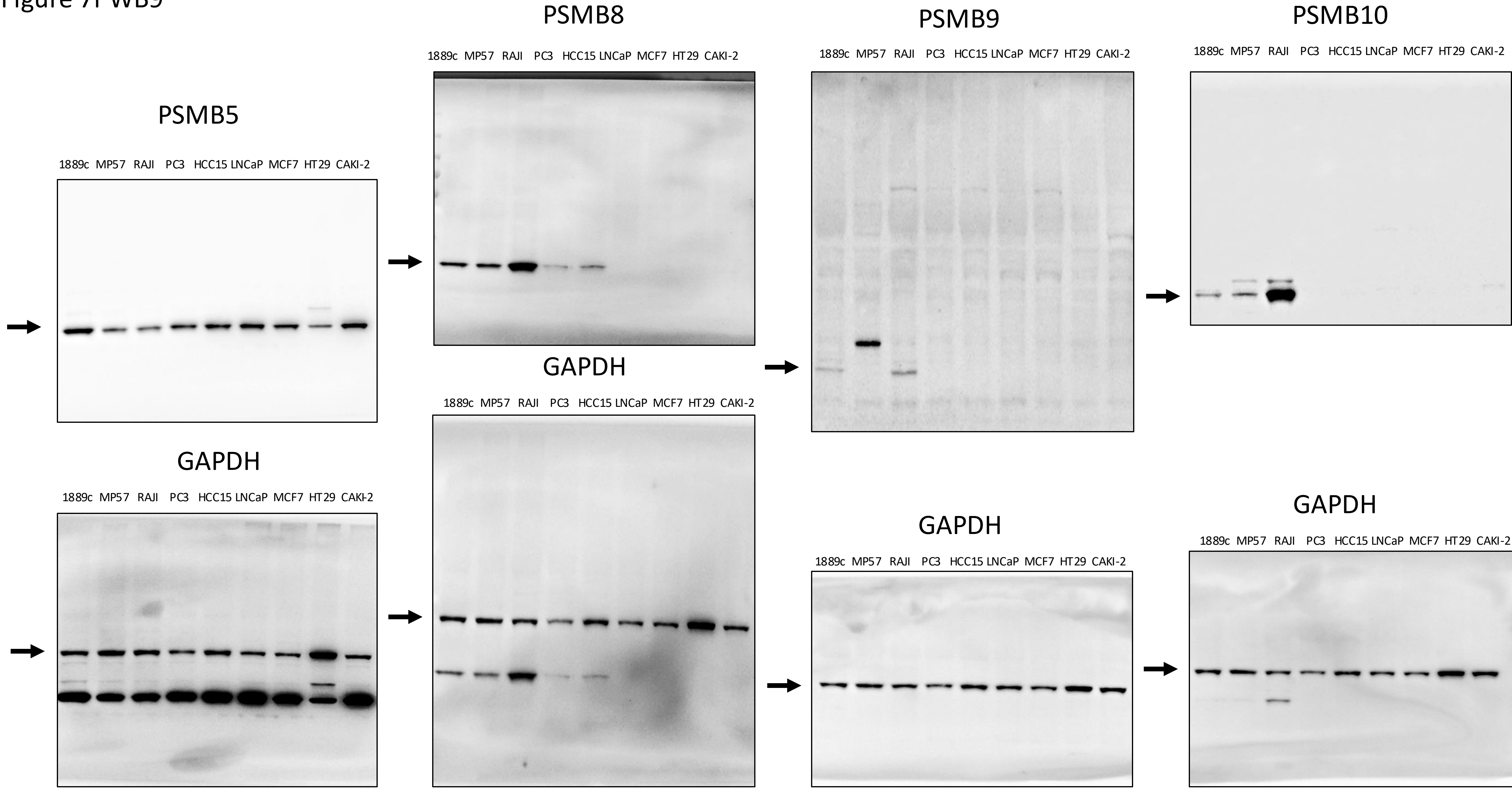

Figure 7f WB10

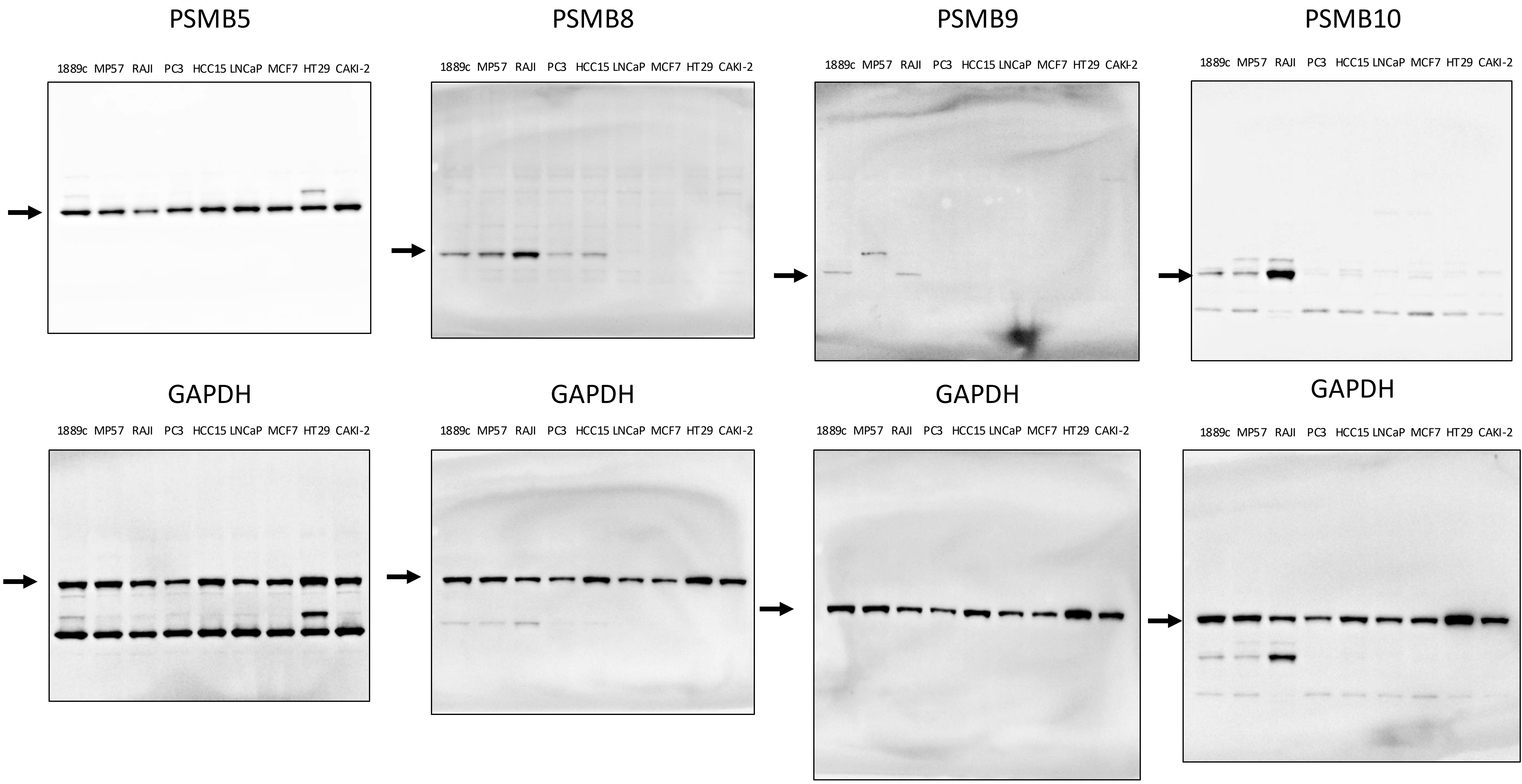

Figure 7f WB11

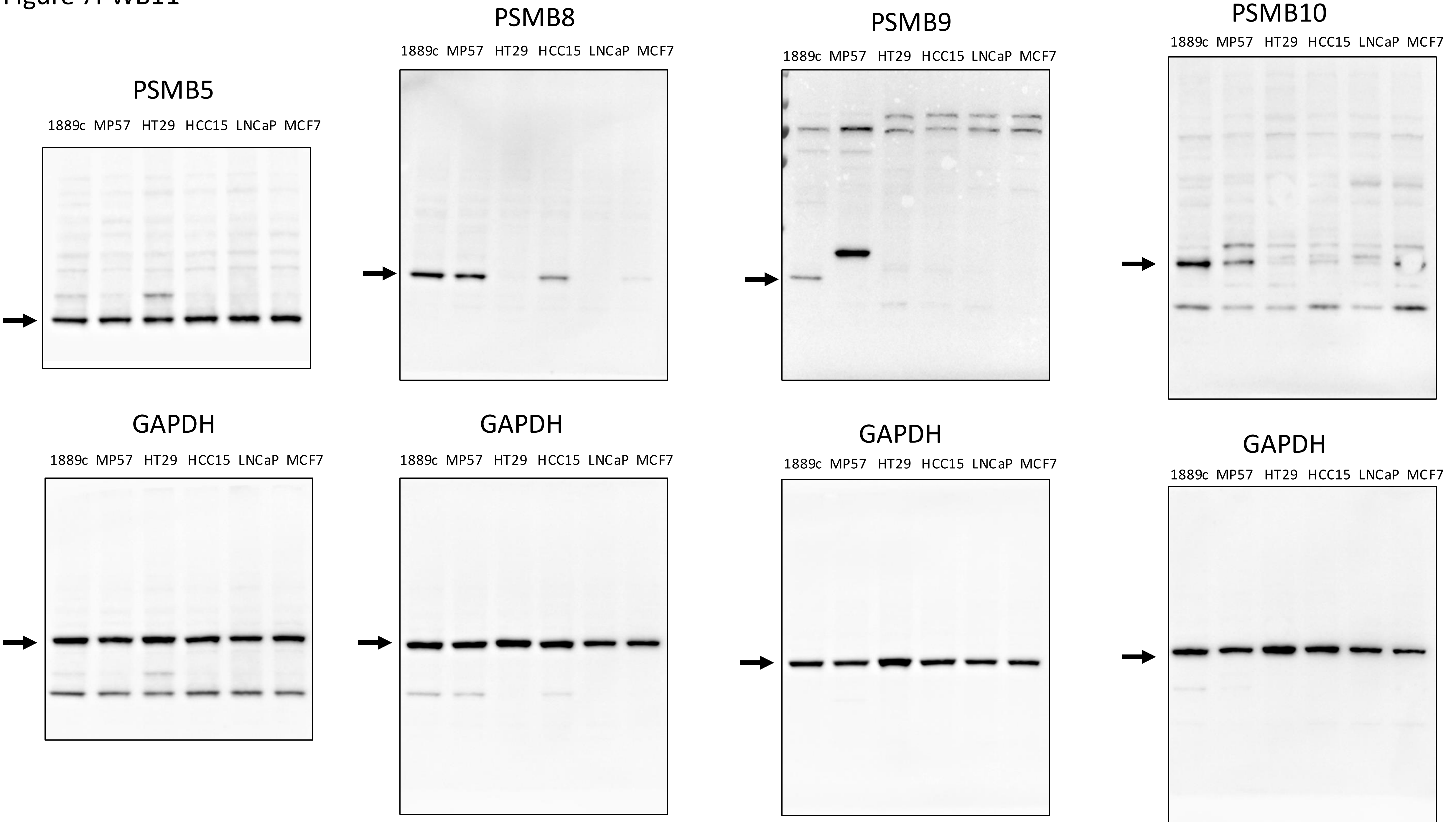

Figure 7f WB12

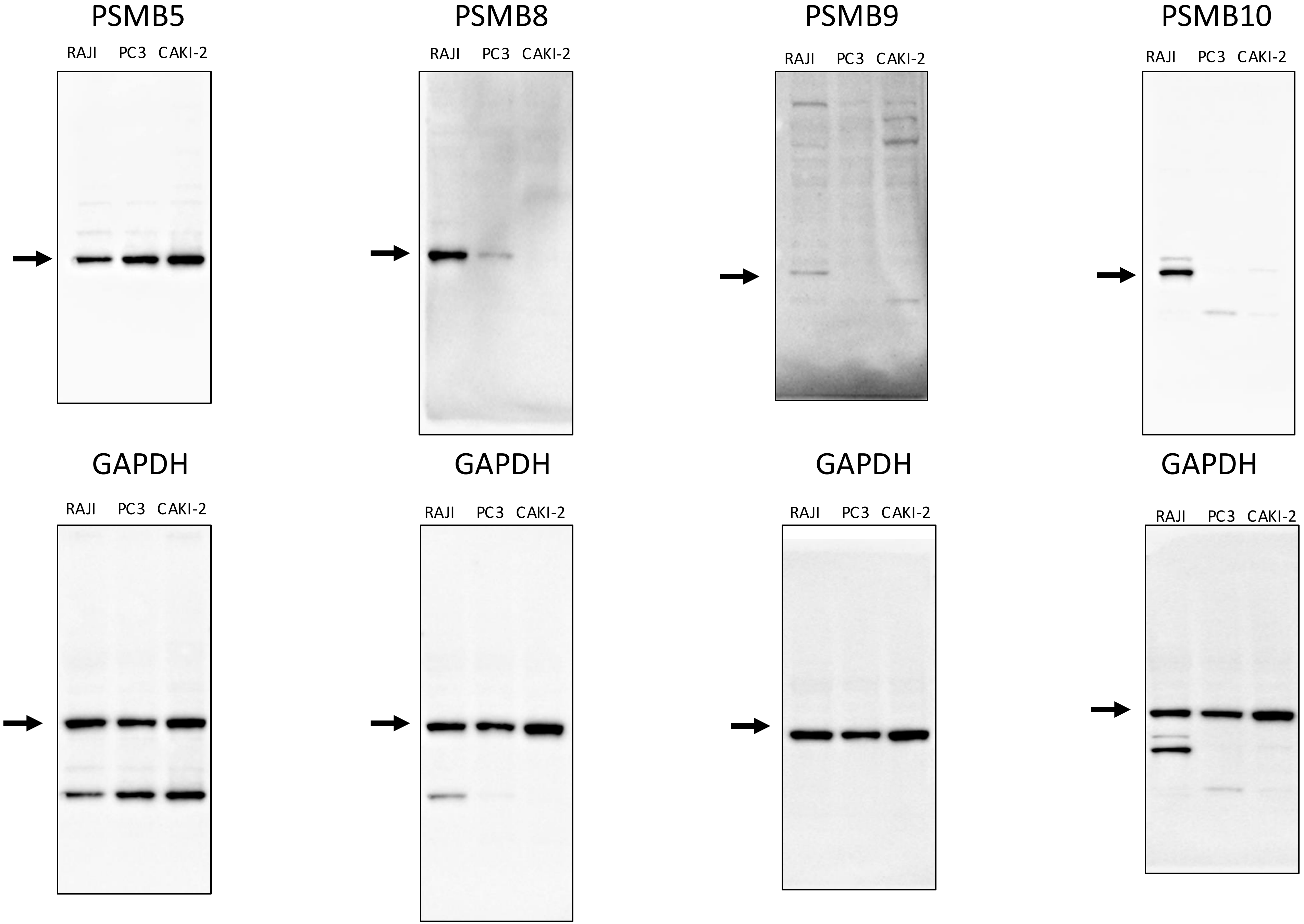

Figure 5D and E WB13

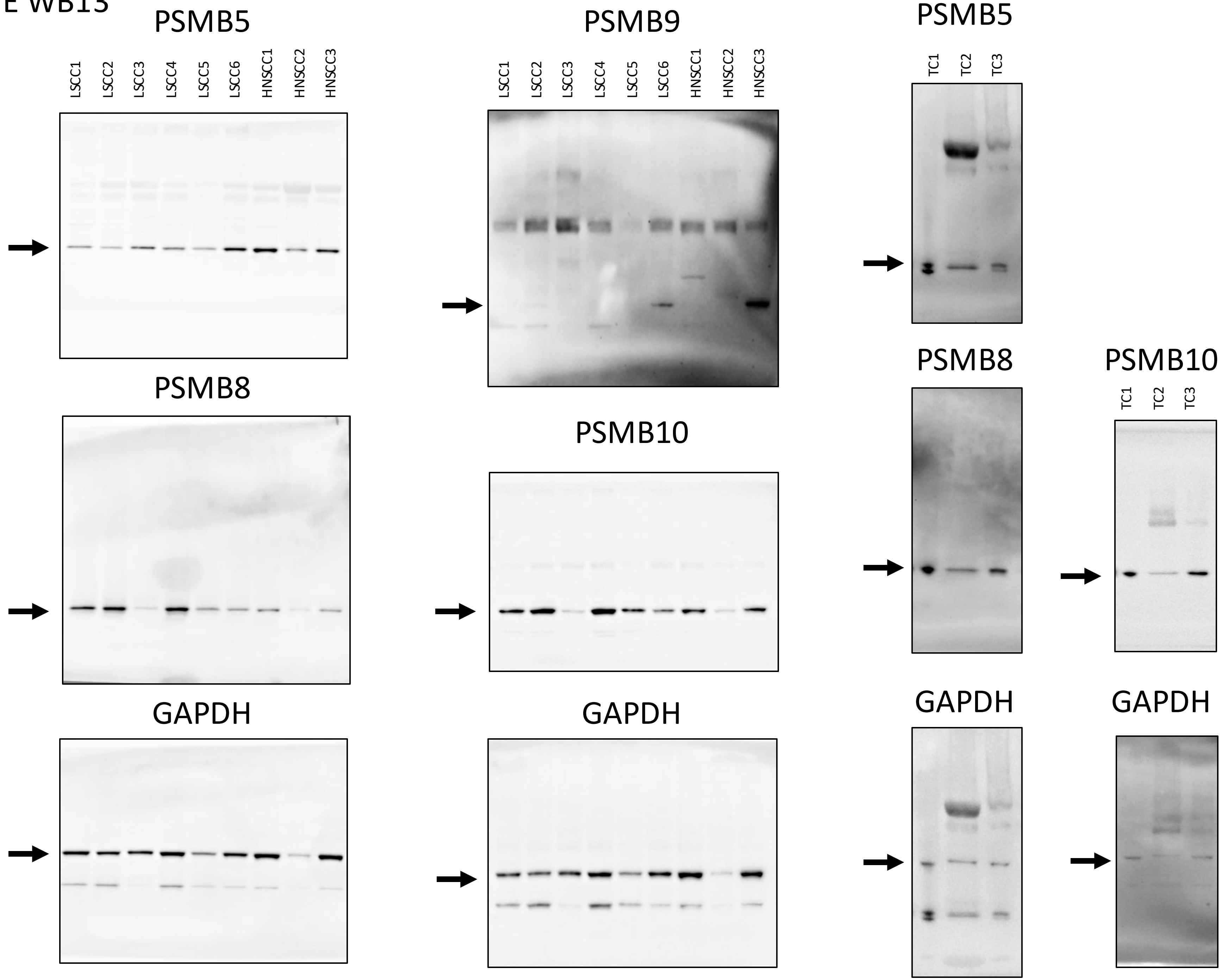

Figure 5D and E WB14

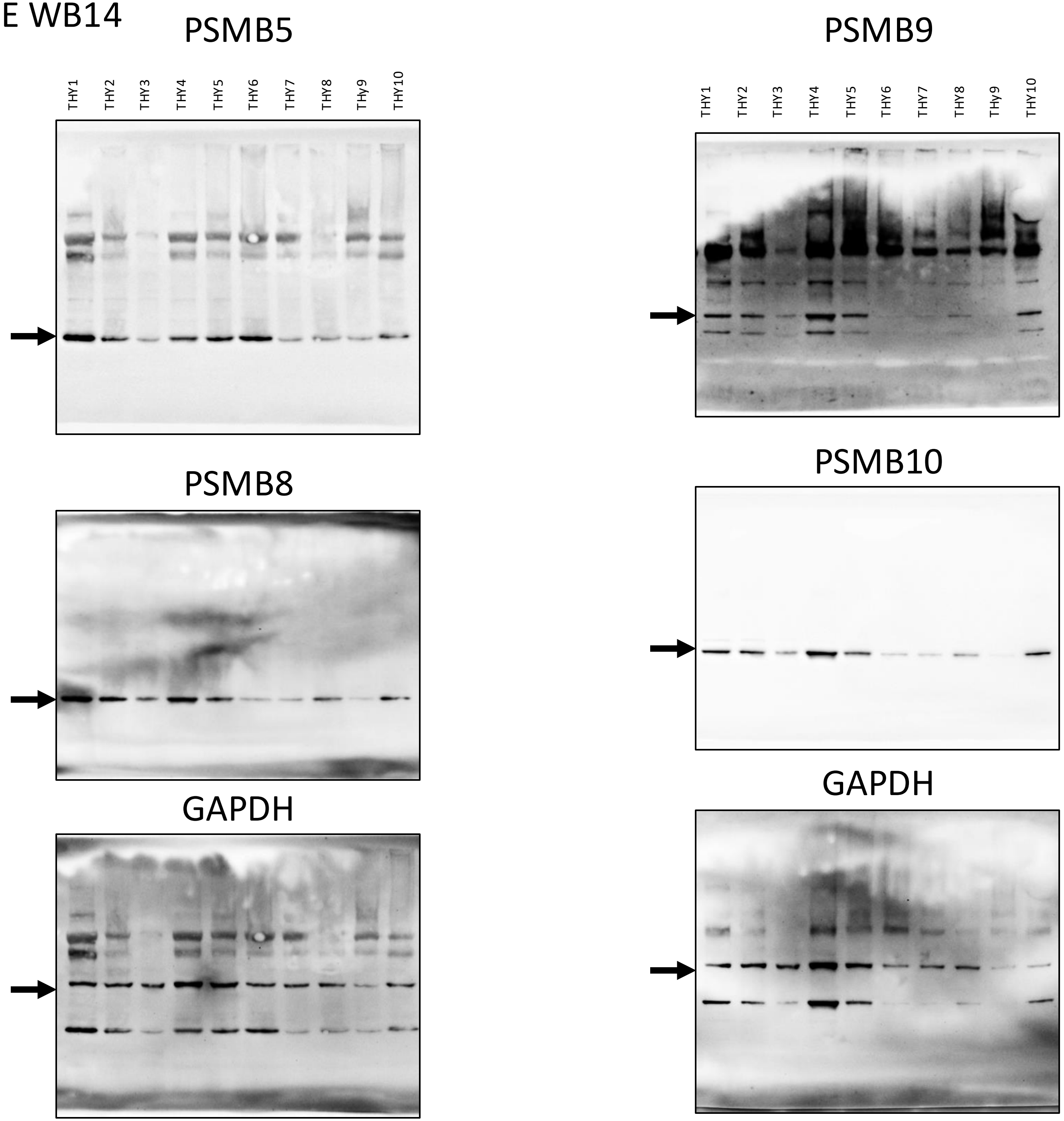

Figure S10C and S11E WB15

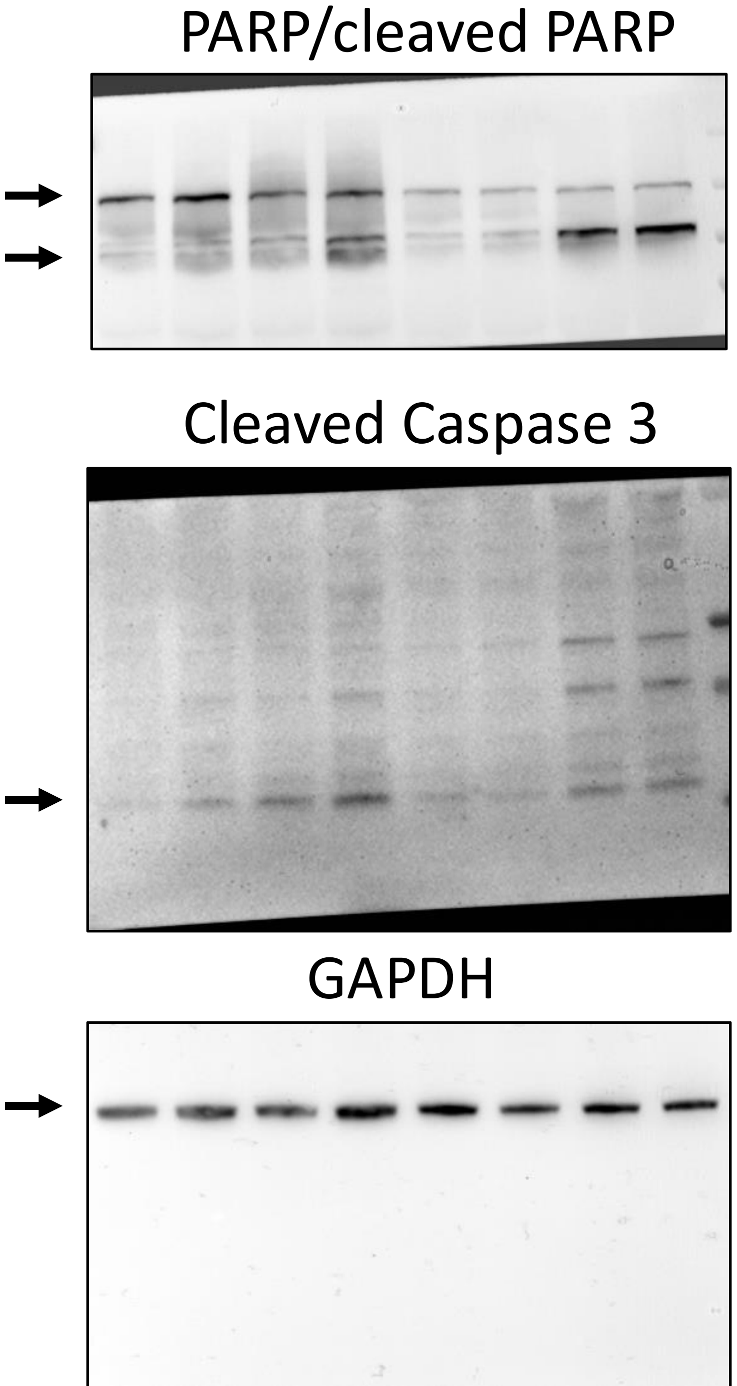

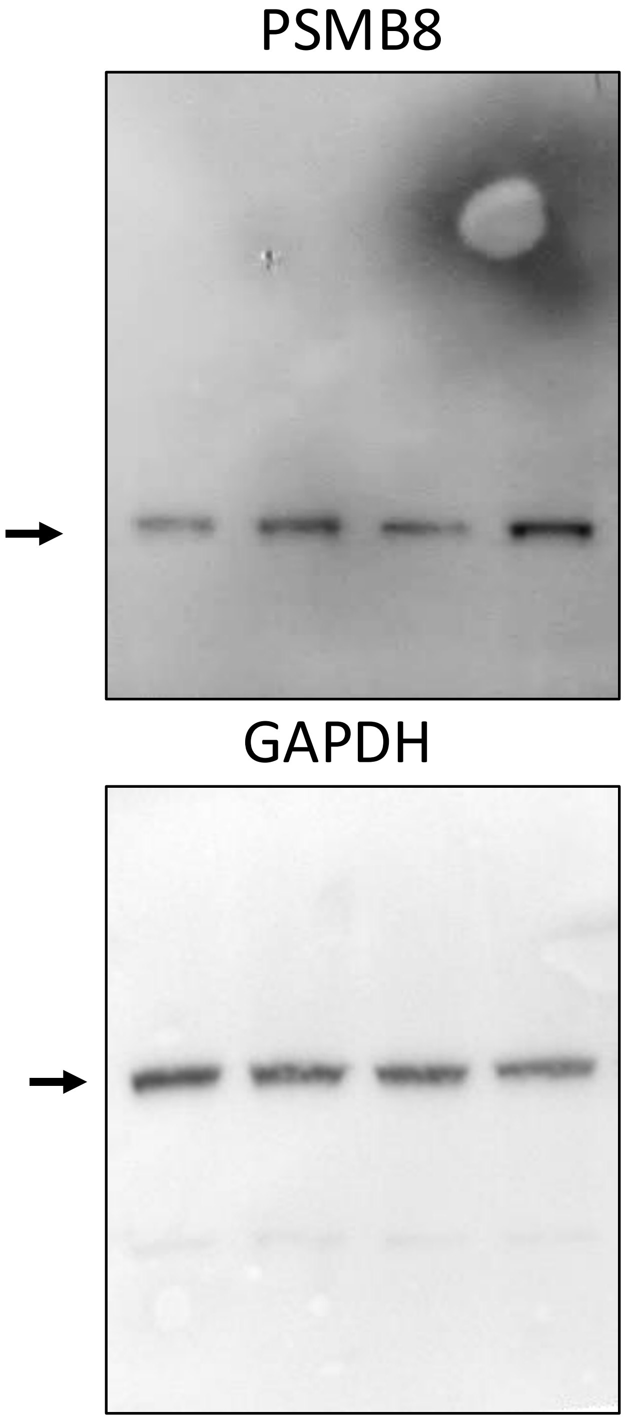

Supplement: Supplementary file 9 — Original Western Blots [file 41419_2025_8240_MOESM9_ESM.pdf]
